# High Conductivity and Diffusion Mechanism of Oxide Ions in Triple Fluorite-Like Layers of Oxyhalides

**DOI:** 10.1021/jacs.4c00265

**Published:** 2024-04-09

**Authors:** Nachi Ueno, Hiroshi Yaguchi, Kotaro Fujii, Masatomo Yashima

**Affiliations:** Department of Chemistry, School of Science, Tokyo Institute of Technology, 2-12-1-W4-17, Ookayama, Meguro-ku, Tokyo 152-8551, Japan

## Abstract

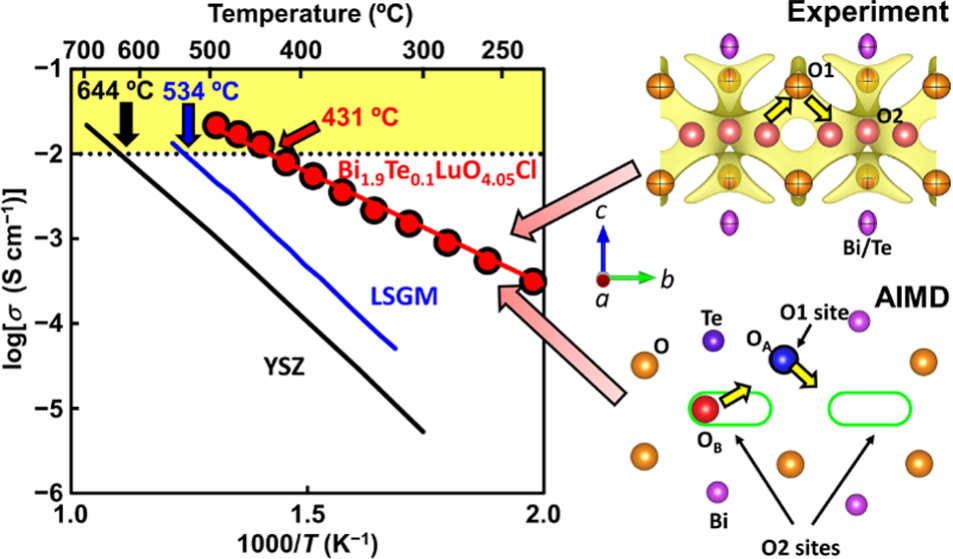

Oxide ion conductors are attractive materials because of their wide range of applications, such as solid oxide fuel cells. Oxide ion conduction in oxyhalides (compounds containing both oxide ions and halide ions) is rare. In the present work, we found that Sillén oxychlorides, Bi_2–*x*_Te_*x*_LuO_4+*x*/2_Cl (*x* = 0, 0.1, and 0.2), show high oxide ion conductivity. The bulk conductivity of Bi_1.9_Te_0.1_LuO_4.05_Cl reaches 10^–2^ S cm^–1^ at 431 °C, which is much lower than 644 °C of yttria-stabilized zirconia (YSZ) and 534 °C of La_0.8_Sr_0.2_Ga_0.83_Mg_0.17_O_2.815_ (LSGM). Thanks to the low activation energy, Bi_1.9_Te_0.1_LuO_4.05_Cl exhibits a high bulk conductivity of 1.5 × 10^–3^ S cm^–1^ even at a low temperature of 310 °C, which is 204 times higher than that of YSZ. The low activation energy is attributed to the interstitialcy oxide ion diffusion in the triple fluorite-like layer, as evidenced by neutron diffraction experiments (Rietveld and neutron scattering length density analyses), bond valence-based energy calculations, static DFT calculations, and ab initio molecular dynamics simulations. The electrical conductivity of Bi_1.9_Te_0.1_LuO_4.05_Cl is almost independent of the oxygen partial pressure from 10^–18^ to 10^–4^ atm at 431 °C, indicating the electrolyte domain. Bi_1.9_Te_0.1_LuO_4.05_Cl also exhibits high chemical stability under a CO_2_ flow and ambient air at 400 °C. The oxide ion conduction due to the two-dimensional interstitialcy diffusion is considered to be common in Sillén oxyhalides with triple fluorite-like layers, such as Bi_1.9_Te_0.1_*R*O_4.05_Cl (*R* = La, Nd, Sm, Eu, Gd, Dy, Ho, Er, Tm, Yb, Lu) and Bi_6–2*x*_Te_2*x*_O_8+*x*_Br_2_ (*x* = 0.1, 0.5). The present study opens a new field of materials chemistry: oxide ion-conducting Sillén oxyhalides with triple fluorite-like layers.

## Introduction

Oxide ion conductors are attracting much attention because of their potential use in applications such as solid oxide fuel cells (SOFCs), oxygen separation membranes, and sensors.^1−13^ Yttria-stabilized zirconia (YSZ) is a commonly used electrolyte in SOFCs. However, the SOFCs with YSZ electrolytes require high operating temperatures (700–1000 °C), which leads to a need to use expensive heat-resistant alloys and degradation, resulting in high costs. In particular, there have been little stable oxide ion conductors exhibiting a high conductivity of 10^–2^ S cm^–1^ below 500 °C. Therefore, it is crucial to explore oxide ion conductors with high conductivity at low temperatures.

Mixed-anion compounds, which contain two or more anions, are of great interest because of the wide variety of structures and properties.^14−16^ The oxyhalides are mixed-anion compounds containing both oxide ions and halide ions that exhibit a variety of material properties such as photocatalysis and magnetic properties.^17−23^ Several oxyhalides such as La_0.9_Sr_0.1_O_0.45_F_2_ and La_0.8_Sr_0.15_Mg_0.05_OBr_0.8_ exhibit halide ion conduction.^24,25^ On the other hand, oxide ion conduction in oxyhalides is rare and therefore worthy of study.^26−28^

In this work, we have synthesized Bi_2–*x*_Te_*x*_LuO_4+*x*/2_Cl (*x* = 0, 0.1, 0.2), Bi_1.9_Te_0.1_*R*O_4.05_Cl (*R* = Nd, Sm, Eu, Gd, Dy, Ho, Er, Tm, Yb), and Bi_6–2*x*_Te_2*x*_O_8+*x*_Br_2_ (*x* = 0.1, 0.5) and investigated their electrical and structural properties. We chose these materials for the following reasons. (1) These materials contain Bi species. It is known that Bi-containing materials, such as Na_0.5_Bi_0.49_Ti_0.98_Mg_0.02_O_2.965_,^29^ Bi_12.5_La_1.5_ReO_24.5_,^30^ Bi_3.9_Sr_0.1_NbO_8–*δ*_Cl,^27^ and CsBi_2_Ti_2_NbO_10–*δ*_,^31^ exhibit high oxide ion conductivities. (2) The parent materials Bi_2_*R*O_4_Cl and Bi_6–2*x*_Te_2*x*_O_8+*x*_Br_2_ (*x* = 0.5) are the Sillén oxyhalides with the triple fluorite-like layers where the interstitial oxygen sites exist, leading to possible interstitialcy oxide ion diffusion. Recently, the oxide ion conduction via the interstitialcy diffusion has attracted attention due to the high conductivity (e.g., hexagonal perovskite-related oxides,^1,32−37^ K_2_NiF_4_-type oxides,^38^ melilite-type oxides,^39,40^ scheelite-type oxides,^41,42^ and mayenite-type oxides^43^). In the present work, Bi_1.9_Te_0.1_LuO_4.05_Cl was found to show high oxide ion conductivity and chemical and electrical stability. The bulk conductivity of Bi_1.9_Te_0.1_LuO_4.05_Cl reaches 10^–2^ S cm^–1^ at 431 °C, which is much lower than 644 °C of (ZrO_2_)_0.92_(Y_2_O_3_)_0.08_ (YSZ) and 534 °C of La_0.8_Sr_0.2_Ga_0.83_Mg_0.17_O_2.815_ (LSGM). Many other compositions with triple fluorite-like layers also showed significant conductivity, suggesting that the series of Sillén oxyhalides with this layer are ionic conductors. The present study opens a new research field for oxide ion conductors and contributes to a better understanding and material diversity.

## Results and Discussion

### Synthesis, Characterization, and Bulk Conductivity of Bi_2–*x*_Te_*x*_LuO_4+*x*/2_Cl

Bi_2–*x*_Te_*x*_LuO_4+*x*/2_Cl samples (*x* = 0, 0.1, 0.2) were prepared by the solid-state reaction method. All observed reflections in the X-ray powder diffraction (XRD) patterns of Bi_2–*x*_Te_*x*_LuO_4+*x*/2_Cl (*x* = 0, 0.1, 0.2) at room temperature were indexed by a single tetragonal Sillén phase (Figure S1a). Electron diffraction patterns of Bi_2–*x*_Te_*x*_LuO_4+*x*/2_Cl (*x* = 0.1) also indicated the tetragonal Sillén phase (Figure S1b, c). Figure S2 shows the lattice parameters of Bi_2–*x*_Te_*x*_LuO_4+*x*/2_Cl as functions of the Te content *x*. The lattice parameter *a* increased, while the *c* parameter decreased with an increase of the Te content *x*, suggesting the formation of solid solutions of Bi_2–*x*_Te_*x*_LuO_4+*x*/2_Cl (*x* = 0.1, 0.2).

**Figure 1:**
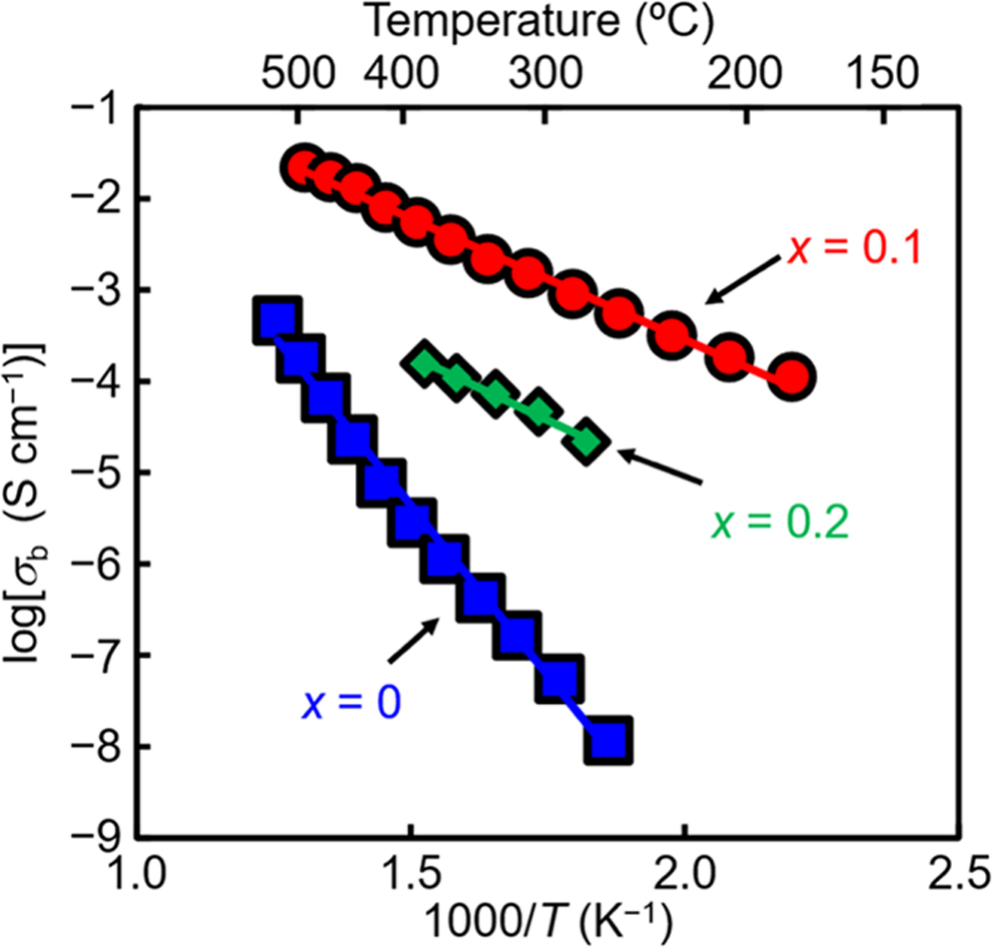
Arrhenius plots of bulk conductivity *σ*_b_ of Bi_2–*x*_Te_*x*_LuO_4+*x*/2_Cl (*x* = 0, 0.1, 0.2) in dry nitrogen. The activation energies for σ_b_ were 1.53(4) eV for *x* = 0, 0.577(12) eV for *x* = 0.1, and 0.61(3) eV for *x* = 0.2.

The bulk conductivity (*σ*_b_) and grain boundary conductivity, (*σ*_gb_) in dry nitrogen were estimated analyzing the AC impedance spectra of Bi_2–*x*_Te_*x*_LuO_4+*x*/2_Cl (*x* = 0, 0.1, 0.2) through the equivalent circuit fitting (Figure 1 and S3–S5, Supplementary Note S1). The capacitance values validated the equivalent circuit fitting. Both the *σ*_b_ and *σ*_gb_ increased with temperature, and the *σ*_b_ was higher than *σ*_gb_ across the entire temperature range (Figure S3). The Te donor-doped material, Bi_1.9_Te_0.1_LuO_4.05_Cl (*x* = 0.1), demonstrated higher *σ*_b_ than that of nondoped Bi_2_LuO_4_Cl (*x* = 0) throughout the whole temperature range (Figure 1). In particular, the *σ*_b_ for *x* = 0.1 was much higher than that for *x* = 0 at low temperatures (e.g., 4.7 × 10^4^ times at 259 °C) because of the lower activation energy for *σ*_b_ of *x* = 0.1 (*E*_a_ = 0.577(12) eV) compared to that of *x* = 0 (*E*_a_ = 1.53(4) eV). The higher conductivity and lower activation energy for the *x* = 0.1 composition were attributable to the excess oxygen content of 0.05 in Bi_1.9_Te_0.1_LuO_4.05_Cl, leading to the interstitialcy oxide ion diffusion as described later. These results were consistent with static DFT calculations, which showed that the Te-doped composition Bi_16_Te_2_Lu_9_O_37_Cl_9_ [= (Bi_1.78_Te_0.22_LuO_4.11_Cl)_9_] exhibited lower activation energy (0.62 eV) compared with the undoped parent material Bi_18_Lu_9_O_36_Cl_9_ [=(Bi_2_LuO_4_Cl)_9_] (1.56 eV) (Supplementary Note S2; Figures S6–S9). The Te-doped composition Bi_16_Te_2_Lu_9_O_37_Cl_9_ showed oxide ion diffusion via the interstitialcy mechanism, while the parent material Bi_18_Lu_9_O_36_Cl_9_ exhibited oxide ion diffusion via the vacancy mechanism and Frenkel defects. Therefore, the lower activation energy *E*_a_ of Bi_1.9_Te_0.1_LuO_4.05_Cl (*x* = 0.1) compared with Bi_2_LuO_4_Cl (*x* = 0.0) was attributable to the oxide ion diffusion via the interstitialcy mechanism.

The bulk conductivity *σ*_b_ for *x* = 0.2 with a higher excess oxygen content of 0.10 was lower than that for *x* = 0.1 with a lower excess oxygen content of 0.05. Such a decrease in the ion conductivity by overdoping has been observed in other ion conductors, possibly due to the clustering and/or defect association among the dopants and oxygen defects.^2,44^ Bi_1.9_Te_0.1_LuO_4.05_Cl (*x* = 0.1) showed the highest *σ*_b_ among the Bi_2–*x*_Te_*x*_LuO_4+*x*/2_Cl materials (*x* = 0, 0.1, 0.2) at 182–492 °C (Figure 1). In particular, the *σ*_b_ of Bi_1.9_Te_0.1_LuO_4.05_Cl was exceptionally high, reaching 1.3 × 10^–2^ S cm^–1^ at 440 °C and 2.2 × 10^–2^ S cm^–1^ at 492 °C, which exceed the threshold value of 10^–2^ S cm^–1^ required for the electrolyte materials of fuel cells.^45^ Based on these results, we focused on our further detailed studies on Bi_1.9_Te_0.1_LuO_4.05_Cl.

Inductively coupled plasma atomic emission spectroscopy (ICP-AES) measurements of Bi_1.9_Te_0.1_LuO_4.05_Cl powders revealed the cation ratio of Bi/Te/Lu = 1.898:0.099:1.004, which agreed well with the ratio of Bi/Te/Lu = 1.9:0.1:1.0 for the nominal composition. X-ray fluorescence (XRF) analysis of Bi_1.9_Te_0.1_LuO_4.05_Cl confirmed the presence of Cl, Bi, Te, and Lu species. Bi_1.9_Te_0.1_LuO_4.05_Cl showed no significant weight changes in the second and third cycles of the thermogravimetric (TG) measurements between 100 and 700 °C in dry nitrogen (Figure S10), indicating no significant changes in the chemical composition and cation valences in this temperature range. The band gap of Bi_1.9_Te_0.1_LuO_4.05_Cl was estimated to be 2.62 eV from the UV–vis diffuse reflectance spectrum, suggesting its nonmetallic nature (Figure S11). The scanning electron microscopy (SEM) image of Bi_1.9_Te_0.1_LuO_4.05_Cl showed the average grain size of 0.39 μm (Figure S12).

**Figure 2:**
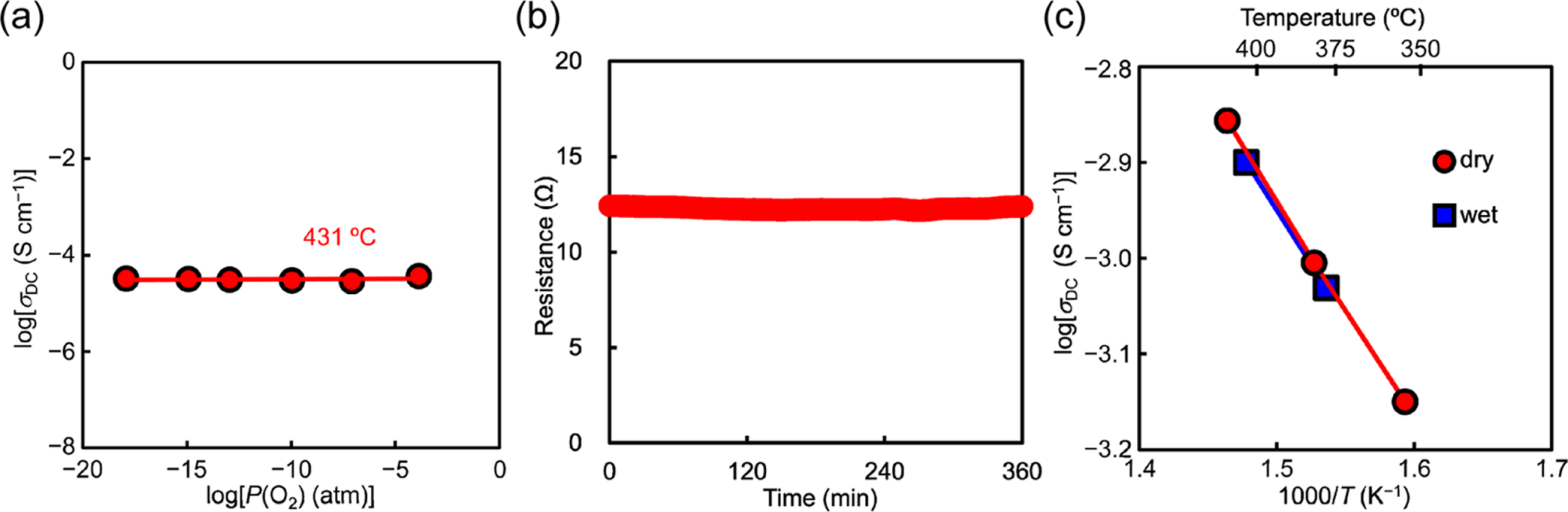
Oxide ion conduction and high chemical and electrical stability of Bi_1.9_Te_0.1_LuO_4.05_Cl. (a) Oxygen partial pressure *P*(O_2_) dependence of the DC electrical conductivity *σ*_DC_ of low-density Bi_1.9_Te_0.1_LuO_4.05_Cl at 431 °C (red closed circles, relative density of 78%). See also Figure S14 and Supplementary Note S3. (b) Time dependence of the resistivity for 1 mA constant current in Bi_1.9_Te_0.1_LuO_4.05_Cl at 600 °C under a dry nitrogen flow (red solid line). (c) Arrhenius plots of *σ*_DC_ of Bi_1.9_Te_0.1_LuO_4.05_Cl in wet (water vapor pressure of 0.021 atm) and dry nitrogen.

As shown in Figure 2a, the direct-current (DC) electrical conductivity *σ*_DC_ of Bi_1.9_Te_0.1_LuO_4.05_Cl was measured by the DC four-probe method and remained almost constant in the oxygen partial pressure *P*(O_2_) range from 1.2 × 10^–18^ to 1.3 × 10^–4^ atm at 431 °C, indicating the electrolyte domain. The total AC electrical conductivity *σ*_total_^AC^ of Bi_1.9_Te_0.1_LuO_4.05_Cl was estimated using the impedance data and remained almost constant in the *P*(O_2_) range from 10^–18^ to 10^–5^ atm at 440 °C (Figure S15b-d), indicating the electrolyte domain, which was consistent with the DC electrical conductivity measurements in Figure 2a. The *σ*_total_^AC^ at *P*(O_2_) = 10^–20^ atm was slightly higher than those in the *P*(O_2_) range from 10^–18^ to 10^–5^ atm, which could be due to the contribution of electronic conduction (Figure S15a). At each *P*(O_2_) of 0.2 and 1 atm, an additional impedance response was observed in the high*-Z*′ region (Figure S15e,f), resulting in the decrease of *σ*_total_^AC^ compared to *σ*_total_^AC^ in the *P*(O_2_) range from 10^–18^ to 10^–5^ atm. The additional impedance response could be due to the partial oxidation. The decrease of conductivity at *P*(O_2_) = 0.2 and 1 atm can be suppressed by using a high-density Bi_1.9_Te_0.1_LuO_4.05_Cl pellet (Figure S14). The XRD pattern of the Bi_1.9_Te_0.1_LuO_4.05_Cl sample after the DC conductivity measurements at different *P*(O_2_) was in agreement with that before the measurements (Figure S13). This indicates no phase decomposition and high chemical stability of the Bi_1.9_Te_0.1_LuO_4.05_Cl sample. The resistance remained almost constant during DC polarization measurements of Bi_1.9_Te_0.1_LuO_4.05_Cl by applying a constant current even at 600 °C (Figure 2b). This suggests no long-range diffusion of the cations and chloride ion in Bi_1.9_Te_0.1_LuO_4.05_Cl. Negligible proton conduction was observed because there was no difference in *σ*_DC_ of Bi_1.9_Te_0.1_LuO_4.05_Cl under wet and dry nitrogen flows (Figure 2c). These findings indicate that the conducting species in Bi_1.9_Te_0.1_LuO_4.05_Cl is oxide ions.

**Figure 3:**
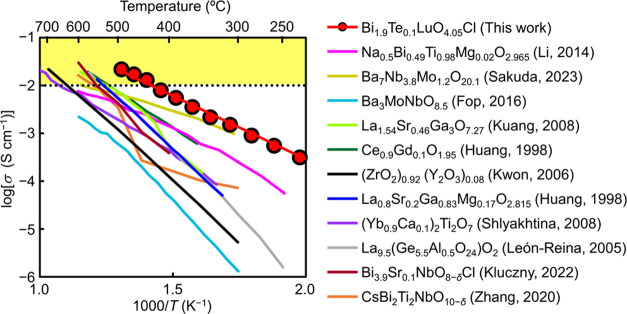
Comparison of the bulk conductivity *σ*_b_ of Bi_1.9_Te_0.1_LuO_4.05_Cl with those of other leading oxide ion conductors: Na_0.5_Bi_0.49_Ti_0.98_Mg_0.02_O_2.965_,^29^ Ba_7_Nb_3.8_Mo_1.2_O_20.1_,^32^ Ba_3_MoNbO_8.5_,^33^ La_1.54_Sr_0.46_Ga_3_O_7.27_,^39^ Ce_0.9_Gd_0.1_O_1.95_,^46^ (ZrO_2_)_0.92_(Y_2_O_3_)_0.08_,^5^ La_0.8_Sr_0.2_Ga_0.83_Mg_0.17_O_2.815_,^47^ (Yb_0.9_Ca_0.1_)_2_Ti_2_O_7_,^48^ La_9.5_(Ge_5.5_Al_0.5_O_24_)O_2_,^49^ Bi_3.9_Sr_0.1_NbO_8–*δ*_Cl,^27^ and CsBi_2_Ti_2_NbO_10–*δ*_.^31^ The black dotted line denotes the conductivity of 0.01 S cm^–1^, and the yellow area stands for the region where the conductivity is higher than 0.01 S cm^–1^.

Figure 3 shows the comparison of the bulk conductivity *σ*_b_ of Bi_1.9_Te_0.1_LuO_4.05_Cl with those of typical oxide ion conductors. It should be noted that the σ_b_ of Bi_1.9_Te_0.1_LuO_4.05_Cl reaches 10^–2^ S cm^–1^ at 431 °C, which is much lower than 644 °C of YSZ and 534 °C of LSGM. At 310 °C, the *σ*_b_ of Bi_1.9_Te_0.1_LuO_4.05_Cl (1.5 × 10^–3^ S cm^–1^) was 204 times higher than that of YSZ (5.3 × 10^–6^ S cm^–1^). At 492 °C, the *σ*_b_ of Bi_1.9_Te_0.1_LuO_4.05_Cl (2.2 × 10^–2^ S cm^–1^) was 3.4 times higher than that of the Ba_7_Nb_3.8_Mo_1.2_O_20.1_ (6.4 × 10^–3^ S cm^–1^).^32^ The *σ*_b_ of Bi_1.9_Te_0.1_LuO_4.05_Cl was comparable to that of Bi_1.9_Te_0.1_LaO_4.05_Cl around 300 °C (Figure S16a).^28^ Additionally, the total conductivity of Bi_1.9_Te_0.1_LuO_4.05_Cl was estimated using the total resistivity in the impedance spectra (Figure S4c,d), as high as 6.8 × 10^–2^ S cm^–1^ at 648 °C and comparable to that of Bi_1.9_Te_0.1_LaO_4.05_Cl in the whole temperature range (Figure S16b).

**Figure 4:**
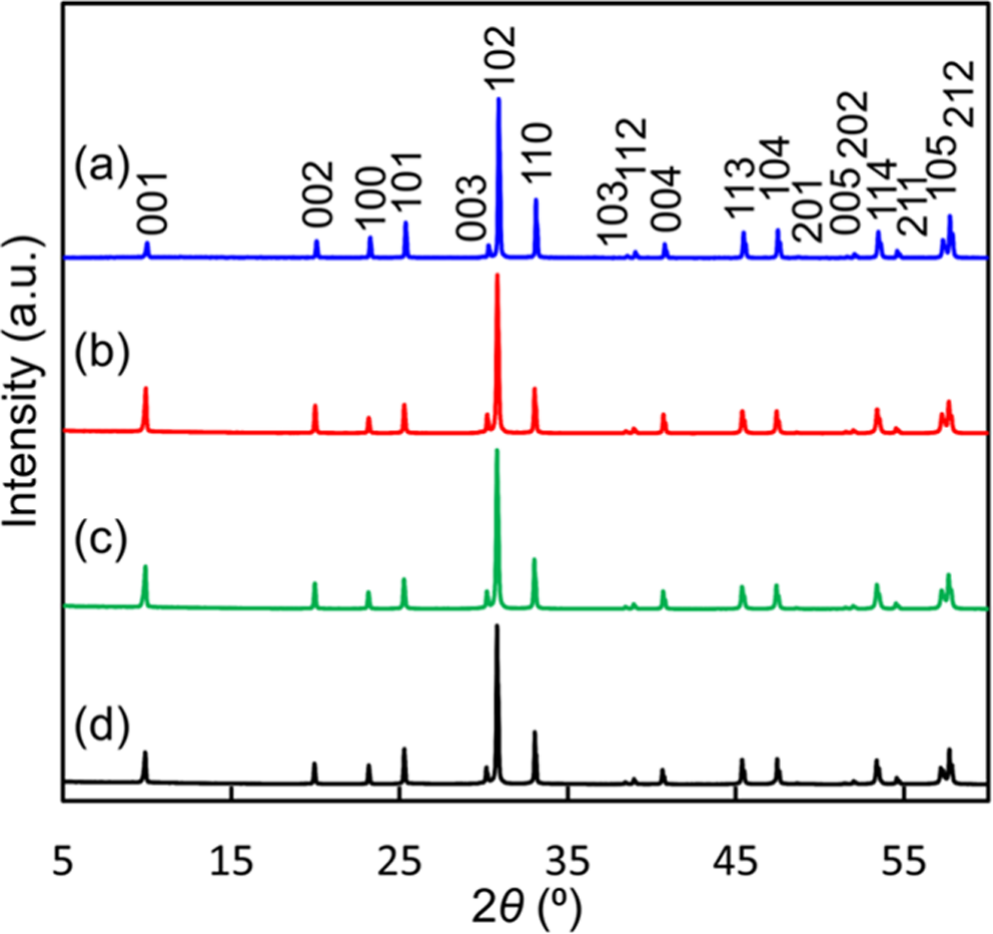
Cu Kα XRD patterns measured at room temperature of Bi_1.9_Te_0.1_LuO_4.05_Cl samples (a) before (blue solid line), (b) after annealing at 400 °C for 100 h in a CO_2_ flow (red solid line), (c) after annealing at 400 °C for 100 h in static air with natural humidity (green solid line), and (d) after annealing at 600 °C for 100 h in static air with natural humidity (black solid line). No decomposition or degradation was observed in all the XRD patterns.

XRD patterns of Bi_1.9_Te_0.1_LuO_4.05_Cl remained unchanged when annealed under a CO_2_ gas flow and static air (approximate water vapor pressure of 1.8 × 10^–3^ atm) (Figure 4), indicating high chemical stability. The reduction of Bi oxides into Bi metal can occur at lower *P*(O_2_) than 10^–13^ atm because the *P*(O_2_) for the Bi/Bi_2_O_3_ equilibrium is of the order of 10^–13^ atm.^50^ In contrast, Bi_1.9_Te_0.1_LuO_4.05_Cl showed a wider electrolyte domain down to the severely reduced atmosphere of *P*(O_2_) = 1.1 × 10^–23^ atm at 450 °C (Figure S17), compared to Bi-based oxides such as Bi_2_(V_0.95_Li_0.05_)O_5.4_ and (Bi_0.8_Er_0.2_)_2_O_3_.^51,52^

Bi_1.9_Te_0.1_LuO_4.05_Cl is a superior oxide ion conductor because of the high oxide ion conductivity and high chemical stability.

### Structural Origins of the High Oxide Ion Conductivity of Bi_1.9_Te_0.1_LuO_4.05_Cl

**Figure 5:**
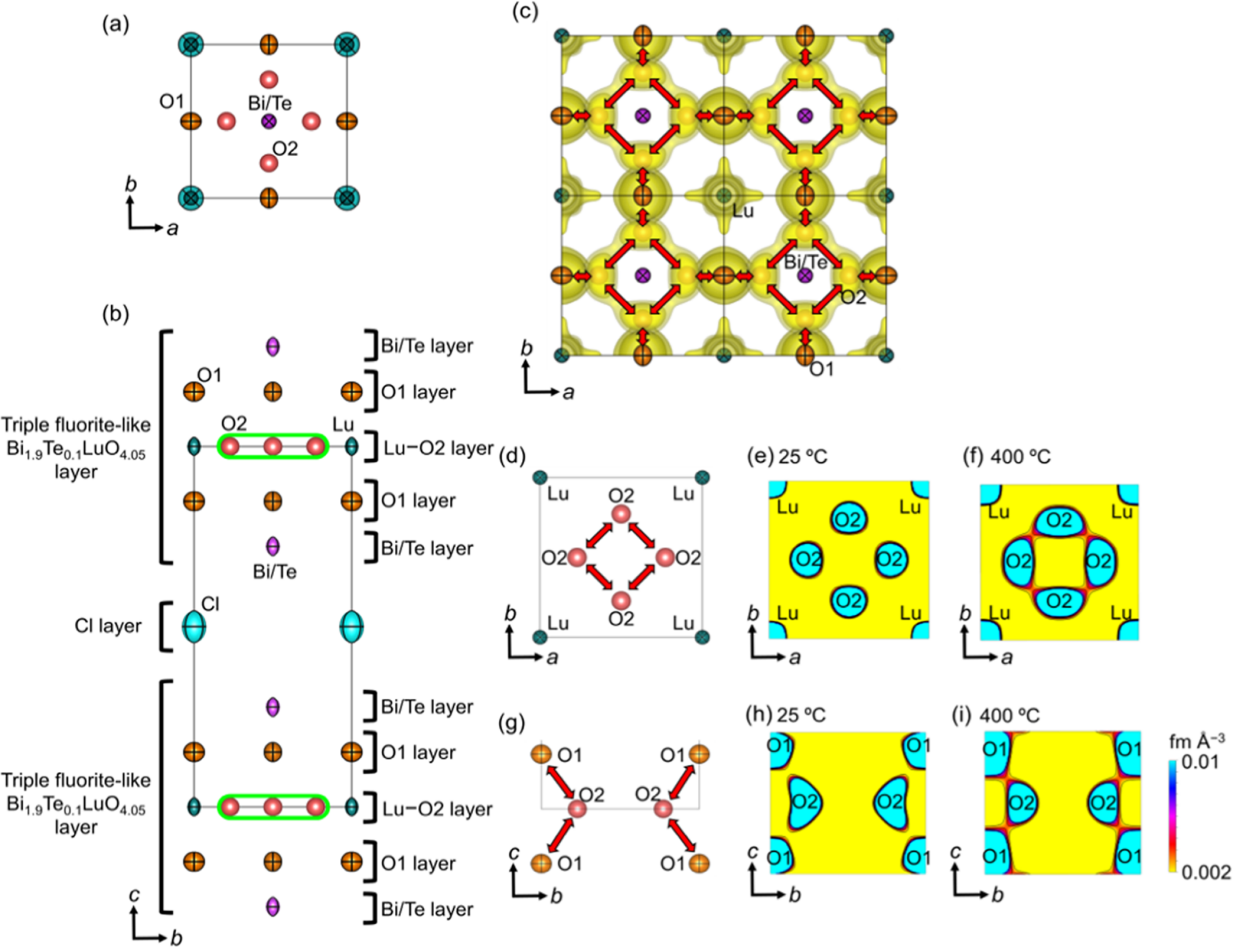
Experimental evidence for the interstitialcy oxide ion diffusion in Bi_1.9_Te_0.1_LuO_4.05_Cl. The refined crystal structure of Bi_1.9_Te_0.1_LuO_4.05_Cl at 400 °C viewed along the *c*-axis [(a) 0.0 ≤  *x*  ≤ 1.0; 0.0 ≤  *y*  ≤ 1.0; – 0.3 ≤  *z*  ≤ 1.3 and (d) for 0.0 ≤ *x* ≤ 1.0; 0 ≤ *y* ≤ 1.0; – 0.1 ≤ *z* ≤ 0.1] and along the *a*-axis [(b) 0.0 ≤ *x* ≤ 1.0; 0.0 ≤ *y* ≤ 1.0; – 0.3 ≤ *z* ≤ 1.3 and (g) for 0.4 ≤ *x* ≤ 0.6; 0 ≤ *y* ≤ 1.0; – 0.2 ≤ *z* ≤ 0.2]. (c) Refined crystal structure of Bi_1.9_Te_0.1_LuO_4.05_Cl at 400 °C viewed along the *c*-axis (0 ≤ *x* ≤ 2.0; 0 ≤ *y* ≤ 2.0; – 0.2 ≤ *z* ≤ 0.2) and corresponding yellow isosurfaces of maximum-entropy method (MEM) neutron scattering length density (NSLD) at 0.002  fm  Å^–3^. MEM NSLD distributions of Bi_1.9_Te_0.1_LuO_4.05_Cl on the *ab*  plane (0 ≤ *x* ≤ 1.0; 0 ≤ *y* ≤ 1.0; *z*  = 0) at (e) 25 °C and (f) 400 °C and on the *bc* plane (*x* = 0.5; 0 ≤ *y* ≤ 1.0; −0.2 ≤ *z* ≤ 0.2) at (h) 25 °C and (i) 400 °C. In (e), (f), (h), and (i), the contour lines are from 0.002 to 0.01 fm  Å^–3^ with the step of 0.0015  fm  Å^–3^. Orange, purple, dark-green, and light-blue ellipsoids denote the lattice O1, Bi/Te, Lu, and Cl atoms, respectively, and red spheres denote interstitial O2 (Table S1). Displacement ellipsoids and spheres are drawn at 75% probability level. The black rectangle in (a) and (b) represents the unit cell. The light-green stadium (rounded rectangle) in (b) represents the interstitial O2 sites. Red arrows in (d) and (g) denote the directions of the oxide ion migration.

The crystal structures of Bi_1.9_Te_0.1_LuO_4.05_Cl and Bi_2_LuO_4_Cl were analyzed using neutron powder diffraction (ND) data measured *in situ* between 25 and 700 °C and at 25 °C, respectively, in order to discuss the origins of the high oxide ion conductivity. The crystal structures of Bi_1.9_Te_0.1_LuO_4.05_Cl and Bi_2_LuO_4_Cl were successfully refined by the Rietveld method based on the tetragonal *P*4/*mmm* Sillén phase in the whole temperature range (Tables S1–S9, Figures S18 and S19). In the parent material Bi_2_LuO_4_Cl, the Rietveld analysis of ND data indicated no interstitial oxygen O2 atoms (Table S9). In contrast, in the Te-doped material Bi_1.9_Te_0.1_LuO_4.05_Cl, the presence of interstitial oxygen O2 atoms was shown by (i) the refined crystal structure, (ii) neutron scattering length density (NSLD) obtained by the maximum entropy method (MEM) analysis, and (iii) bond valence-based energy landscape (O2 in Figure 5; see the details in Supplementary Note S4 and Figures S20–S22, Tables S1–S9). The calculated bond valence sums (BVSs) agreed with the averaged formal charges (Tables S1 and S9). The refined crystal parameters of Bi_1.9_Te_0.1_LuO_4.05_Cl from the synchrotron XRD data at 27 °C agreed with those from the ND data at 25 °C (Supplementary Note S5, Tables S1 and S10, Figure S23). The crystal structure of Bi_1.9_Te_0.1_LuO_4.05_Cl was also confirmed by the high-resolution transmission electron microscopy (HRTEM) image (Figure S24). These results indicated the validity of the refined crystal structures of Bi_1.9_Te_0.1_LuO_4.05_Cl and Bi_2_LuO_4_Cl. The lattice parameters increased with an increase of temperature (Figure S25), and the average thermal expansion coefficients along the *a*- and *c*-axes between 25 and 700 °C were 1.4983(5) × 10^–5^ and 2.3387(8) × 10^–5^ K^–1^, respectively (Supplementary Note S6).

The refined crystal structure of Bi_1.9_Te_0.1_LuO_4.05_Cl and Bi_2_LuO_4_Cl is composed of a triple fluorite-like layer and a Cl layer, indicating the Sillén phase (Figure S26, and Supplementary Note S7). As Bi_2_LuO_4_Cl has no interstitial oxygen O2 atoms (Figure S26a), the formation of Frenkel defects (vacancies at the O1 site and interstitial O2 atoms) is needed for oxide ion migration via the vacancy mechanism in Bi_2_LuO_4_Cl, leading to high activation energy for bulk conductivity *σ*_b_ due to the high formation energy of Frenkel defects (2.63 eV) and low *σ*_b_ at low temperatures (Supplementary Note S2). In sharp contrast, Bi_1.9_Te_0.1_LuO_4.05_Cl has interstitial oxygen O2 atoms (O2 in Figures 5 and S26b); thus, the oxide ions in Bi_1.9_Te_0.1_LuO_4.05_Cl can migrate via the interstitialcy mechanism, leading to low activation energy for bulk conductivity *σ*_b_. The interstitial O2 atoms make the coordination number of Lu atoms higher (Figure S27). Therefore, the flexibility of the coordination of the Lu atom may be important for the formation of interstitial O2 atoms. Since the triple fluorite-like layer has the interstitial oxygen site, interstitialcy oxide ion diffusion via the lattice O1 and interstitial O2 sites is enabled in the fluorite-like layer of Bi_1.9_Te_0.1_LuO_4.05_Cl (Figure 5), which is the key for the high oxide ion conductivity of Bi_1.9_Te_0.1_LuO_4.05_Cl. In contrast, other known Bi-containing oxide ion conductors, such as Aurivillius-phase Bi_2_V_0.9_Cu_0.1_O_5.35_ and the Sillén–Aurivillius compound Bi_3.9_Sr_0.1_NbO_8–*δ*_Cl, have double fluorite-like layers without interstitial oxygen sites.^27,53^ Therefore, other Bi-containing oxide ion conductors can only show oxide ion diffusion by the conventional vacancy mechanism. The interstitialcy oxide ion diffusion in the triple fluorite-like layer is a very unique feature of Bi_1.9_Te_0.1_LuO_4.05_Cl.

The oxygen atom at the interstitial O2 site of Bi_1.9_Te_0.1_LuO_4.05_Cl was localized at 25 °C (Figure 5e,h), while it exhibited larger spatial distributions at a higher temperature of 400 °C (Figure 5f,i), which corresponded to the higher oxide ion conductivity at 400 °C (Figure 1). It is worth mentioning that the NSLD distributions were connected between the lattice O1 and interstitial O2 sites and between the two adjacent interstitial O2 sites at 400 °C (Figure 5f,i), which was consistent with the bond valence-based energy (BVE) landscapes in Bi_1.9_Te_0.1_LuO_4.05_Cl at 400 °C (Figure S22a,b). This observation indicated two-dimensional (2D) oxide ion diffusion paths through both the lattice O1 and interstitial O2 sites in the triple fluorite-like layer, which invoked the interstitialcy diffusion mechanism (Figure 5c,f,i). In the interstitialcy diffusion mechanism, an oxide ion at the interstitial site migrates to a lattice site by knocking a neighboring lattice atom to an adjacent interstitial site. Interstitialcy migrations were observed in other ion conductors, such as Bi_1.9_Te_0.1_LaO_4.05_Cl,^28^ Pr_2_Ni_0.75_Cu_0.25_O_4+*δ*_,^54^ Ba_7_Nb_3.8_Mo_1.2_O_20.1_,^32^ La_2.2_Sr_0.8_F_4.2_S_2_,^16^ La_0.9_Sr_0.1_O_0.45_F_2_,^24^ and Ba_0.6_La_0.4_F_2.4_.^55^ From the BVE landscapes in Bi_1.9_Te_0.1_LuO_4.05_Cl at 400 °C (Figure S22), the energy barrier for oxide ion migration along the *a*- and *b*-axes was estimated to be 0.22 eV, which was significantly lower than that along the *c*-axis (2.24 eV), supporting the 2D oxide ion migration in the triple fluorite-like layer.

**Figure 6:**
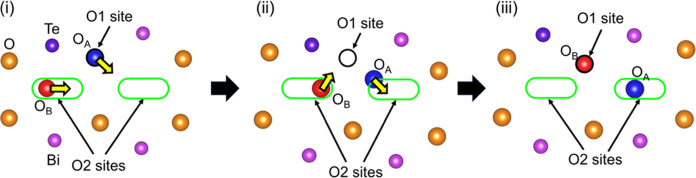
Snapshots of oxide ion migration in the AIMD simulations of Bi_16_Te_2_Lu_9_O_37_Cl_9_ at 1100 °C. Elapsed times are (i) 0, (ii) 80, and (iii) 280 fs. Orange, blue, red, dark-purple, and purple spheres denote the lattice oxygen atoms, O_A_ atom, O_B_ atom, and Te and Bi atoms, respectively. Yellow arrows denote the directions of the oxide ion migration. The black circle and light-green stadium (rounded rectangle) denote the O1 and O2 sites, respectively.

Ab initio molecular dynamics (AIMD) simulations of Bi_16_Te_2_Lu_9_O_37_Cl_9_ (3 × 3 × 1 supercell) were performed at 1100 °C to investigate the local dynamics and oxide ion diffusion. Similar to the experimental observation (Figure 5f,i), the oxide ion migrated through both the lattice O1 and interstitial O2 sites in the triple fluorite-like layer (Figure 6, Supplementary Video). O_A_ and O_B_ atoms migrated to adjacent sites between 0 and 280 fs as follows. At the initial state (i), O_B_ and O_A_ oxide ions existed at the interstitial O2 and lattice O1 sites, respectively. Through the transition state (ii), at the final state (iii), O_B_ and O_A_ were located at the same lattice O1 and adjacent interstitial O2 sites, respectively. An interstitial oxide ion O_B_ (red sphere) at the O2 site pushes (kicks) another oxide ion O_A_ (blue sphere) at the nearest-neighbor lattice O1 site toward an adjacent vacant interstitial O2 site, clearly showing cooperative migration of oxide ions via the interstitialcy mechanism (Figure 6, Supplementary Video). A similar oxide ion diffusion mechanism was also reported for Bi_1.9_Te_0.1_LaO_4.05_Cl.^28^ Therefore, this mechanism is considered to be a common feature of the Sillén phase with a triple fluorite-like layer.

### Synthesis and Electrical Conductivity of Oxyhalides with Triple Fluorite-like Layers

**Figure 7:**
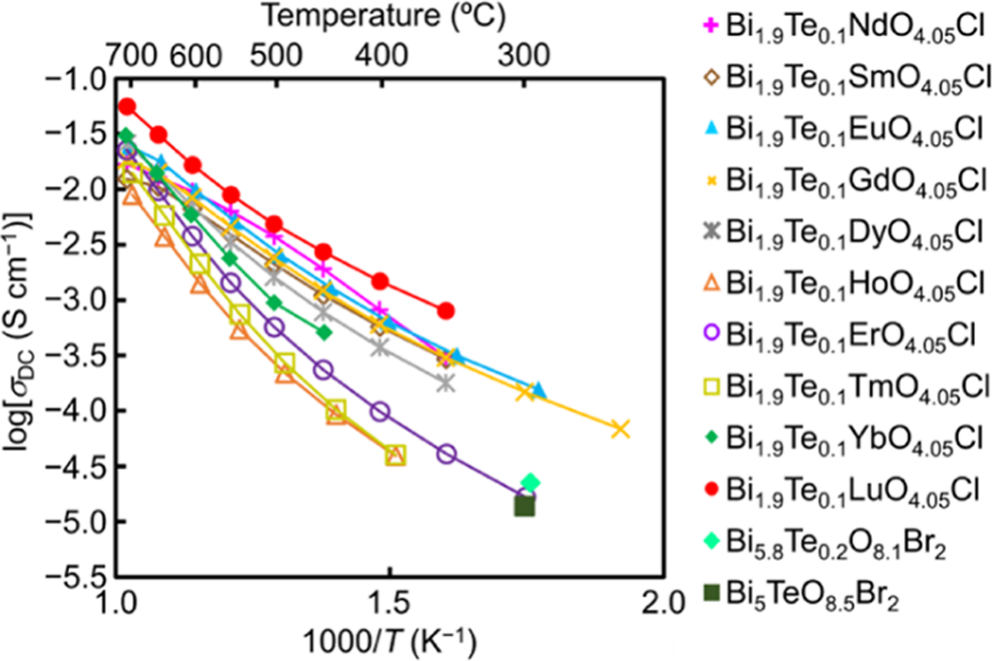
Arrhenius plots of DC electrical conductivity *σ*_DC_ of Bi_1.9_Te_0.1_*R*O_4.05_Cl (*R* = Nd, Sm, Eu, Gd, Dy, Ho, Er, Tm, Yb, Lu) and Bi_6–2*x*_Te_2*x*_O_8+*x*_Br_2_ (*x* = 0.1, 0.5) in dry nitrogen. At any temperature, Bi_1.9_Te_0.1_LuO_4.05_Cl exhibited the highest *σ*_DC_ among these materials (e.g., 5.1 × 10^–2^ S cm^–1^ at 707 °C).

The present study has demonstrated that the high oxide ion conduction in Bi_1.9_Te_0.1_LuO_4.05_Cl is ascribed to the oxide ion migration in the triple fluorite-like layer via the interstitialcy mechanism. Therefore, the triple fluorite-like layer in the Sillén phase is the key to the high oxide ion conductivity. The triple fluorite-like layer has been prevalently found in a number of oxyhalides including Bi_5_TeO_8.5_Br_2_,^56^ Bi_5_TeO_8.5_I_2_,^57^ YSb_2_O_4_*X* (*X* = Cl, Br),^58^ Sm_1.3_Sb_1.7_O_4_Cl,^59^ Sm_1.5_Sb_1.5_O_4_Br,^59^ and Bi_2_*R*O_4_*X* (*R* = Y, La–Lu; *X* = Cl, Br, I).^60^ In this work, we synthesized new materials Bi_1.9_Te_0.1_*R*O_4.05_Cl (*R* = Nd, Sm, Eu, Gd, Dy, Ho, Er, Tm, Yb, Sc) and Bi_6–2*x*_Te_2*x*_O_8+*x*_Br_2_ (*x* = 0.1). A known oxybromide Bi_6–2*x*_Te_2*x*_O_8+*x*_Br_2_ (*x* = 0.5) was also prepared. XRD patterns showed the single Sillén phase for Bi_1.9_Te_0.1_*R*O_4.05_Cl (*R* = Nd, Sm, Eu, Gd, Dy, Ho, Er, Tm, Yb) and the formation of the Sillén phase for Bi_6–2*x*_Te_2*x*_O_8+*x*_Br_2_ (*x* = 0.1, 0.5) (Figure S28). The Sillén phase was not obtained for Bi_1.9_Te_0.1_ScO_4.05_Cl. Therefore, we performed preliminary electrical conductivity measurements of Bi_1.9_Te_0.1_*R*O_4.05_Cl (*R* = Nd, Sm, Eu, Gd, Dy, Ho, Er, Tm, Yb) and Bi_6–2*x*_Te_2*x*_O_8+*x*_Br_2_ (*x* = 0.1, 0.5) in dry nitrogen and found their high electrical conductivities (Figure 7). The BVE landscapes indicated that the oxide ion diffusion in the triple fluorite-like layers occurs also in many Sillén phases such as Bi_2_NdO_4_Cl, Bi_2_GdO_4_Cl, and Bi_5_TeO_8.5_Br_2_ (Figure S29). These results suggest that many oxyhalides with the triple fluorite-like layer are high oxide ion conductors.

## Conclusions

Oxide ion conduction in oxyhalides is rare. Here, we have discovered high oxide ion conductors, Bi-containing Sillén oxyhalides, Bi_2–*x*_Te_*x*_LuO_4+*x*/2_Cl (*x* = 0, 0.1, and 0.2). Bi_1.9_Te_0.1_LuO_4.05_Cl exhibits a high bulk conductivity of 2.2 × 10^–2^ S cm^–1^ at 492 °C, the electrolyte domain in the oxygen partial pressure range from 1.2 × 10^–18^ to 1.3 × 10^–4^ atm at 431 °C, and high chemical stability under CO_2_ at 400 °C and static air at 600 and 400 °C. The bulk conductivity of Bi_1.9_Te_0.1_LuO_4.05_Cl is as high as 10^–2^ S cm^–1^ at 431 °C, which is much lower than 644 °C of YSZ and 534 °C of LSGM. These results indicate that Bi_1.9_Te_0.1_LuO_4.05_Cl is a superior oxide ion conductor. The two-dimensional oxide ion migration by the interstitialcy mechanism via the lattice O1 and interstitial O2 sites in the triple fluorite-like layer was indicated by high-temperature ND studies (Rietveld analyses and neutron scattering length density distributions), bond valence-based energy calculations, static DFT calculations, and ab initio molecular dynamics simulations. The activation energy for the bulk conductivity *E*_a_ of the parent material Bi_2_LuO_4_Cl is high (*E*_a_ = 1.53(4) eV) probably due to the high formation energy of Frenkel defects, leading to low conductivity at low temperatures. In sharp contrast, in the Te-doped compositions (*x* = 0.1 and 0.2 in Bi_2–*x*_Te_*x*_LuO_4+*x*/2_Cl), the activation energy is low (e.g., *E*_a_ = 0.577(12) eV for *x* = 0.1) due to the presence of significant amounts of carrier (interstitial oxygen O2 atoms) without the Frenkel defects, resulting in high conductivity at low temperatures. Therefore, the low activation energy and high conductivity of Bi_1.9_Te_0.1_LuO_4.05_Cl are attributed to the interstitialcy mechanism for oxide ion diffusion due to the presence of interstitial O2 atoms. The interstitialcy mechanism in the triple fluorite-like layer is observed not only in Bi_1.9_Te_0.1_LuO_4.05_Cl but also in Bi_1.9_Te_0.1_LaO_4.05_Cl,^28^ suggesting that the concerted interstitialcy mechanism for oxide ion diffusion is a common feature in Sillén oxyhalides with triple fluorite-like layers. There are still many other Sillén oxyhalides with triple fluorite-like layers. The present study has demonstrated that some of them also exhibit high electrical conductivity, suggesting high oxide ion conductivity. Further investigation of Sillén oxyhalides with triple fluorite-like layers will lead to the discovery of superior oxide ion conductors. The present study opens a new field for oxide ion conductors: oxide ion-conducting Sillén oxyhalides with triple fluorite-like layers.

## Materials and Methods

### Synthesis of Bi_2–*x*_Te_*x*_LuO_4+*x*/2_Cl Samples

The Bi_2–*x*_Te_*x*_LuO_4+*x*/2_Cl samples (*x* = 0, 0.1, and 0.2) were synthesized by a solid-state reaction method. The starting materials, Lu_2_O_3_, Bi_2_O_3_, TeO_2_, and BiOCl (99.9% purity), were mixed and ground with an agate mortar for 30 min. The mixtures were pressed into pellets at 200 MPa by cold isostatic pressing, followed by sintering at 800 °C for 24 h in a vacuum quartz tube. The resulting sintered pellets were used for electrical conductivity measurements. Powder samples obtained by crushing and grinding of the sintered pellets were used for X-ray powder diffraction (XRD), ICP-AES, thermogravimetric (TG), and UV–vis diffuse reflectance (UV–vis) measurements.

### Synthesis of Bi_1.9_Te_0.1_*R*O_4.05_Cl (*R* = Nd, Sm, Eu, Gd, Dy, Ho, Er, Tm, Yb) and Bi_6–2*x*_Te_2*x*_O_8+*x*_Br_2_ (*x* = 0.1, 0.5) Samples

Bi_1.9_Te_0.1_*R*O_4.05_Cl (*R* = Nd, Sm, Eu, Gd, Dy, Ho, Er, Tm, Yb, Sc) and Bi_6–2*x*_Te_2*x*_O_8+*x*_Br_2_ (*x* = 0.1, 0.5) samples were also prepared by the solid-state reaction method. The starting materials, *R*_2_O_3_, Bi_2_O_3_, TeO_2_, and BiOCl, were used to prepare Bi_1.9_Te_0.1_*R*O_4.05_Cl (*R* = Nd, Sm, Eu, Gd, Dy, Ho, Er, Tm, Yb, Sc), while Bi_2_O_3_, TeO_2_, and BiOBr were utilized for the preparation of Bi_6–2*x*_Te_2*x*_O_8+*x*_Br_2_ (*x* = 0.1, 0.5). The starting materials were mixed and ground with an agate mortar for 30 min. The mixtures were pressed into pellets at 200 MPa by cold isostatic pressing and then sintered at 750 to 800 °C for 24–36 h in a vacuum quartz tube. The resulting sintered pellets were used for electrical conductivity measurements. Powder samples obtained by crushing and grinding of the sintered pellets were used for XRD measurements.

### Characterization of the Samples

Cu Kα XRD data of Bi_2–*x*_Te_*x*_LuO_4+*x*/2_Cl (*x* = 0, 0.1, 0.2), Bi_1.9_Te_0.1_*R*O_4.05_Cl (*R* = Nd, Sm, Eu, Gd, Dy, Ho, Er, Tm, Yb, Sc), and Bi_6–2*x*_Te_2*x*_O_8+*x*_Br_2_ (*x* = 0.1, 0.5) samples were measured at room temperature using a MiniFlex600 diffractometer (Rigaku Co. Ltd.) to identify the existing phases. Cu Kα XRD data of Bi_2–*x*_Te_*x*_LuO_4+*x*/2_Cl (*x* = 0, 0.1, 0.2) with the internal standard silicon were also taken at 32 °C with a Rigaku RINT2550 diffractometer to determine the accurate lattice parameters. The chemical composition of Bi_1.9_Te_0.1_LuO_4.05_Cl was obtained by inductively coupled plasma atomic emission spectroscopy (ICP-AES). X-ray fluorescence (XRF) analysis of Bi_1.9_Te_0.1_LuO_4.05_Cl was performed using a Rigaku NEX DE. TG analyses of Bi_1.9_Te_0.1_LuO_4.05_Cl were carried out in a dry nitrogen flow from 50 to 700 °C with heating and cooling rates of 10 °C min^–1^ using a Bruker-AXS TG-DTA2020SA. The heating and cooling process was repeated three times in the TG measurements. The band gap of Bi_1.9_Te_0.1_LuO_4.05_Cl was estimated from the UV–vis diffuse reflectance spectrum (JASCO V-650, 200–800 nm). The scanning electron microscopy (SEM) images of Bi_1.9_Te_0.1_LuO_4.05_Cl were taken using a KEYENCE VE-8800 SEM after annealing the sample at 810 °C in an Ar flow. Selected-area electron diffraction (SAED) patterns and HRTEM images of Bi_1.9_Te_0.1_LuO_4.05_Cl were observed using a transmission electron microscope, JEM-2010, operated at an accelerating voltage of 200 kV. To investigate the chemical stability, Bi_1.9_Te_0.1_LuO_4.05_Cl samples were annealed (1) at 400 °C for 100 h in a CO_2_ flow, (2) at 400 °C for 100 h in static air with natural humidity, and (3) at 600 °C for 100 h in static air with natural humidity. Cu Kα XRD data of the annealed samples were measured at room temperature using the MiniFlex600 diffractometer.

### Electrical Conductivity Measurements

Pt electrodes were attached to the sintered pellets of Bi_2–*x*_Te_*x*_LuO_4+*x*/2_Cl (*x* = 0, 0.1, 0.2) (diameter: 3.8 mm, thickness: 8.0 mm, relative density: 94%), which were used for the impedance measurements. The impedance spectra were recorded under a dry nitrogen atmosphere using a Solartron 1260 impedance analyzer (10 MHz–0.1 Hz, alternating voltage of 100 mV). The measured impedance spectra were analyzed using *ZView* software (Scribner Associates, Inc.) to extract the bulk conductivity *σ*_b_ and grain boundary conductivity *σ*_gb_. The direct-current (DC) electrical conductivity (*σ*_DC_) measurements of Bi_1.9_Te_0.1_LuO_4.05_Cl were performed at 431 and 450 °C under various oxygen partial pressures *P*(O_2_) by the DC four-probe method using a sintered pellet (diameter: 4.4 mm, thickness: 13.6 mm, relative density: 78% at 431 °C; diameter: 3.8 mm, thickness: 12.2 mm, relative density: 97% at 450 °C). The total AC electrical conductivity (*σ*_total_^AC^) was estimated using the impedance data at 440 °C under various oxygen partial pressures *P*(O_2_) using a sintered pellet (diameter: 4.1 mm, thickness: 10.7 mm, relative density: 97%). The *P*(O_2_) was controlled by an oxygen pump (STLab Co., Ltd.; SiOC-200CB) to obtain the *P*(O_2_) dependence of the electrical conductivity of Bi_1.9_Te_0.1_LuO_4.05_Cl. To investigate the chemical stability, the Cu Kα XRD data of Bi_1.9_Te_0.1_LuO_4.05_Cl samples before and after the conductivity measurements at different *P*(O_2_) were measured at room temperature. The DC polarization measurements were performed using a sintered pellet of Bi_1.9_Te_0.1_LuO_4.05_Cl (diameter: 17.5 mm, height: 1.3 mm, relative density: 88%) by applying a constant current (1 mA) in a dry nitrogen atmosphere for 6 h at 600 °C. We attempted to measure the oxide ion transport number using an oxygen concentration cell but were unable to obtain reasonable data due to the reactions between Bi_1.9_Te_0.1_LuO_4.05_Cl and glass seals.

### Neutron Diffraction Measurements and Crystal Structure Analyses

Neutron powder diffraction (ND) data of Bi_2–*x*_Te_*x*_LuO_4+*x*/2_Cl (*x* = 0, 0.1, 0.2) were measured with the time-of-flight (TOF) neutron diffractometers iMATERIA^61^ and SuperHRPD^62,63^ (MLF, J-PARC). Rietveld analyses of the ND and XRD data were carried out using the *Z*-code program.^64^ The neutron scattering length density (NSLD) distribution of Bi_1.9_Te_0.1_LuO_4.05_Cl was calculated by the maximum-entropy method (MEM) using *Z-MEM*(65) based on the structure factors obtained in the Rietveld analysis. Bond valence-based energy (BVE)^66^ landscapes for an oxide ion O^2–^ in the crystal structure of Bi_1.9_Te_0.1_LuO_4.05_Cl at 400 °C, Bi_2_NdO_4_Cl, Bi_2_GdO_4_Cl, and Bi_5_TeO_8.5_Br_2_ at room temperature were calculated using SoftBV^67^ (approximate spatial resolution: 0.01 Å). *VESTA 3* was used to depict the crystal structures, NSLD distributions, and BVE landscapes.^68^

### Static Density Functional Theory (DFT) Calculations and Ab Initio Molecular Dynamics (AIMD) Simulations

The generalized gradient approximation (GGA) electronic calculations were performed using the Vienna Ab initio Simulation Package (VASP).^69^ The oxide ion diffusion was examined by static DFT calculations and ab initio molecular dynamics (AIMD) simulations. Atomic coordinates of Bi_18_Lu_9_O_36_Cl_9_ (3 × 3 × 1 supercell) and Bi_16_Te_2_Lu_9_O_37_Cl_9_ (3 × 3 × 1 supercell) were optimized in the space group *P*1 where the lattice parameters were fixed to the experimental values at 32 °C. For DFT calculations, the projector augmented-wave (PAW) potentials for Lu, Bi, Te, O, and Cl atoms, plane-wave basis sets with a cutoff of 500 eV, and Perdew–Burke–Ernzerhof (PBE) GGA functionals were used.^70^ A 3 × 3 × 3 set of *k*-point meshes was used in the Monkhorst–Pack scheme. To investigate the vacancy and interstitialcy oxide ion migration, the relaxation of atom positions with the convergence criterion of 0.01 eV Å^–1^ was performed by moving an O anion step by step.

AIMD simulations of Bi_16_Te_2_Lu_9_O_37_Cl_9_ (= Bi_1.78_Te_0.22_LuO_4.11_Cl, 3 × 3 × 1 supercell) were performed using VASP with the GGA-PBE functional. AIMD simulations were carried out at a constant temperature of 1100 °C for 80 ps with a time step of 2 fs within the canonical (NVT) ensemble using a Nosé thermostat, after the heating process (1 °C fs^–1^) within the microcanonical (NVE) ensemble. To reduce the computational cost, the reciprocal space integration was carried out only at the Γ point and the cutoff energy was set to 400 eV. The AIMD snapshots were visualized with the OVITO program.^71^

## Supporting Information Available

XRD patterns; lattice parameters; Arrhenius plots; complex impedance plane plots; equivalent circuits; notes; energy profiles and corresponding atomic arrangements; thermogravimetric data; Tauc plot; SEM micrograph; oxygen partial pressure dependence of the DC electrical conductivity; refined crystallographic parameters and reliability factors; Rietveld patterns; refined crystal structures; MEM NSLD distributions; BVE landscapes; and coordination polyhedra (PDF)
Migration of oxide ions via the interstitialcy mechanism obtained by AIMD simulations (MP4)
