# Supplementary material for: High Conductivity and Diffusion Mechanism of Oxide Ions in Triple Fluorite-Like Layers of Oxyhalides
Source: J Am Chem Soc. 2024 Apr 9;146(16):11235–44. doi: 10.1021/jacs.4c00265 (PMC11046479; doi:10.1021/jacs.4c00265)
Supplement: Supplementary file 1 — ja4c00265_si_001.pdf [file ja4c00265_si_001.pdf]

Supporting Information

Title

High Conductivity and Diffusion Mechanism of Oxide Ions in Triple Fluorite-Like Layers of Oxyhalides

Authors

Nachi Ueno, Hiroshi Yaguchi, Kotaro Fujii, Masatomo Yashima*

* Corresponding author: yashima@cms.titech.ac.jp

Affiliations

Department of Chemistry, School of Science, Tokyo Institute of Technology, 2-12-1-W4-17, Ookayama, Meguro-ku, Tokyo 152-8551, Japan

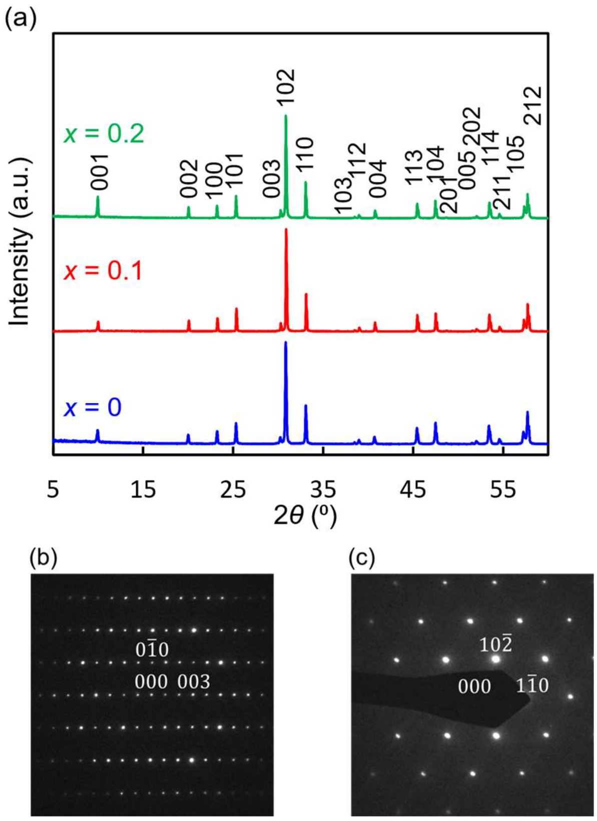

Figure S1. (a) Cu Kα X-ray powder diffraction (XRD) patterns of $Bi_{2-x}Te_xLuO_{4+x/2}Cl$ (x = 0, 0.1, 0.2) samples at room temperature. Electron diffraction patterns of the $Bi_{1.9}Te_{0.1}LuO_{4.05}Cl$ sample along the (b) [100] and (c) [221] zone axes. The *hkl* denotes the reflection index based on the primitive tetragonal lattice (*P*4/*mmm* Sillén phase).

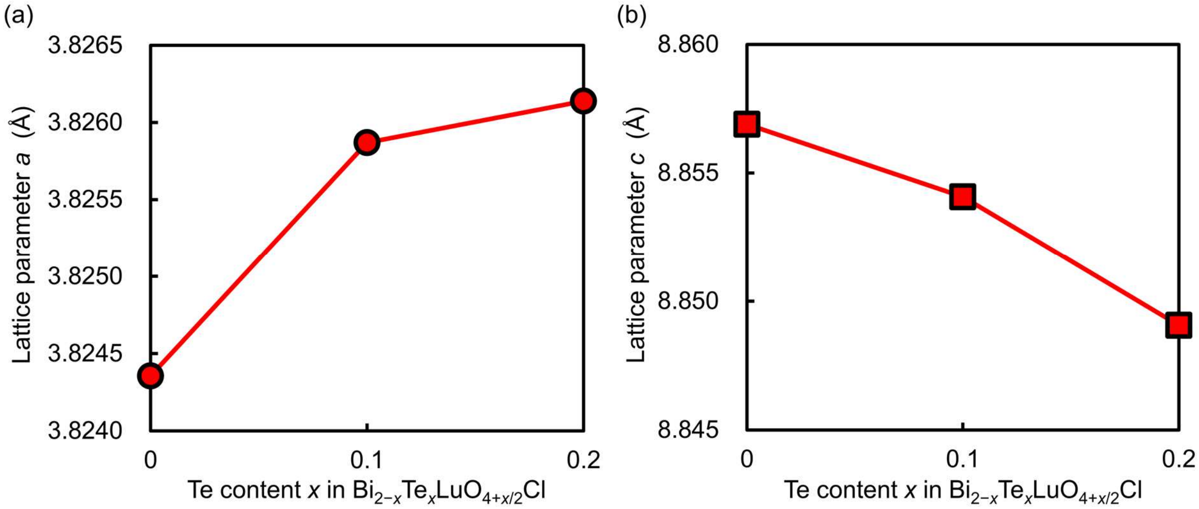

Figure S2. Composition x dependence of the lattice parameters (a) a and (b) c of $Bi_{2-x}Te_xLuO_{4+x/2}Cl$ (x = 0, 0.1, 0.2) at 32 °C.

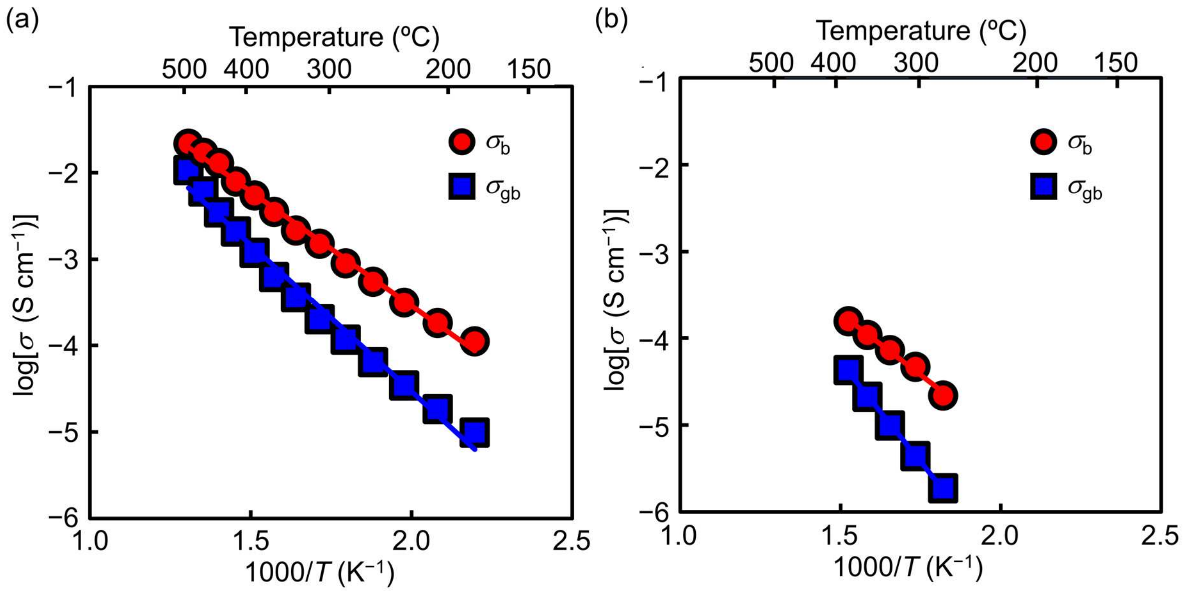

Figure S3. Arrhenius plots of the bulk conductivity (red closed circles and red solid line, σ_b) and grain-boundary conductivity (blue closed squares and blue solid line, σ_{gb}) in dry nitrogen of $Bi_{2-x}Te_xLuO_{4+x/2}Cl$ ((a) x = 0.1 and (b) x = 0.2). The activation energies for σ_{gb} were 0.961(19) eV for x = 0.1 and 1.54(3) eV for x = 0.2, which were higher than those for σ_b (0.577(12) eV for x = 0.1 and 0.61(3) eV for x = 0.2).

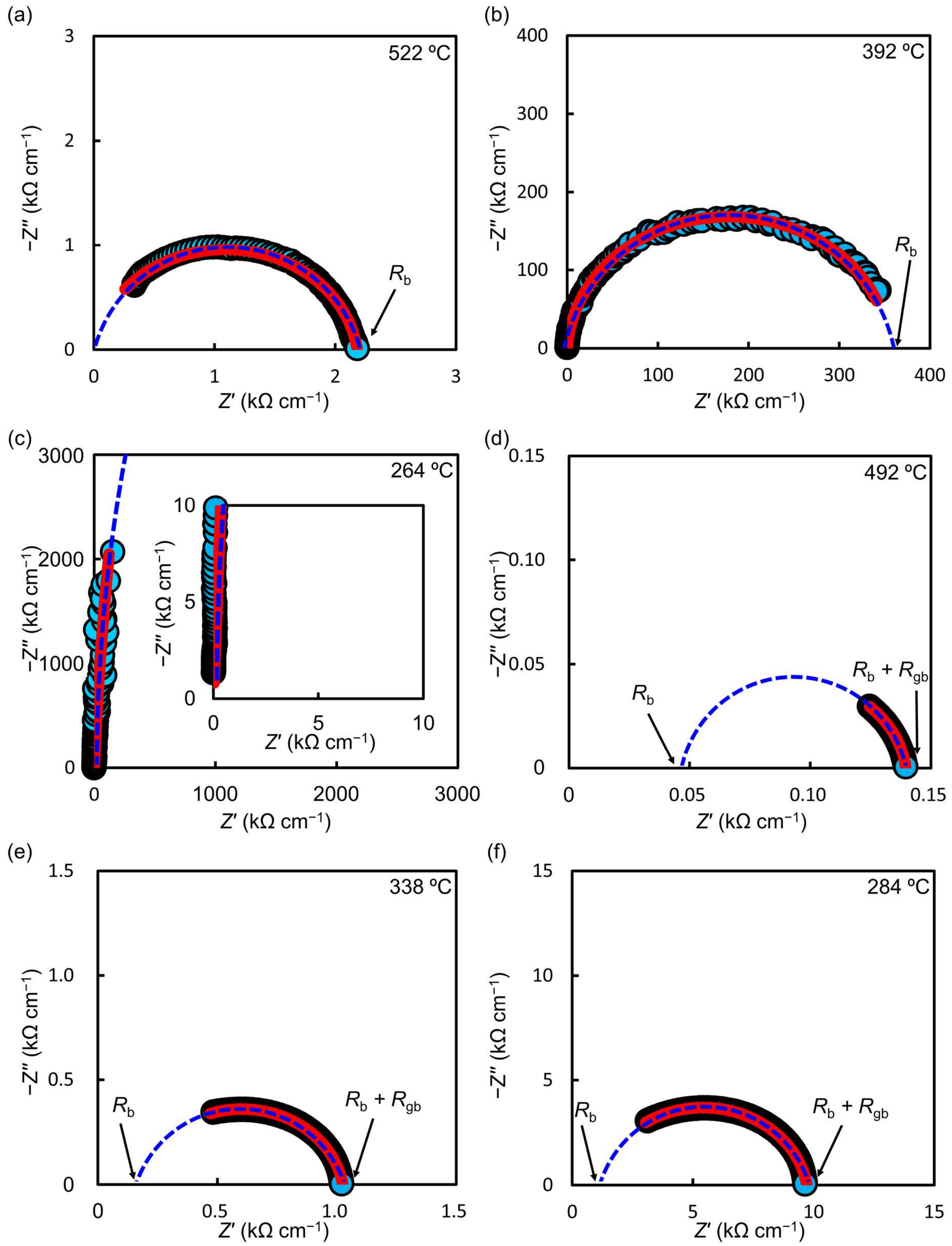
(a)
522 ºC
R_b
−Z″ (kΩ cm−1)
Z′ (kΩ cm−1)
(b)
392 ºC
R_b
(c)
264 ºC
(d)
492 ºC
R_b
$R_b + R_{gb}$
(e)
338 ºC
R_b
$R_b + R_{gb}$
(f)
284 ºC
R_b
$R_b + R_{gb}$

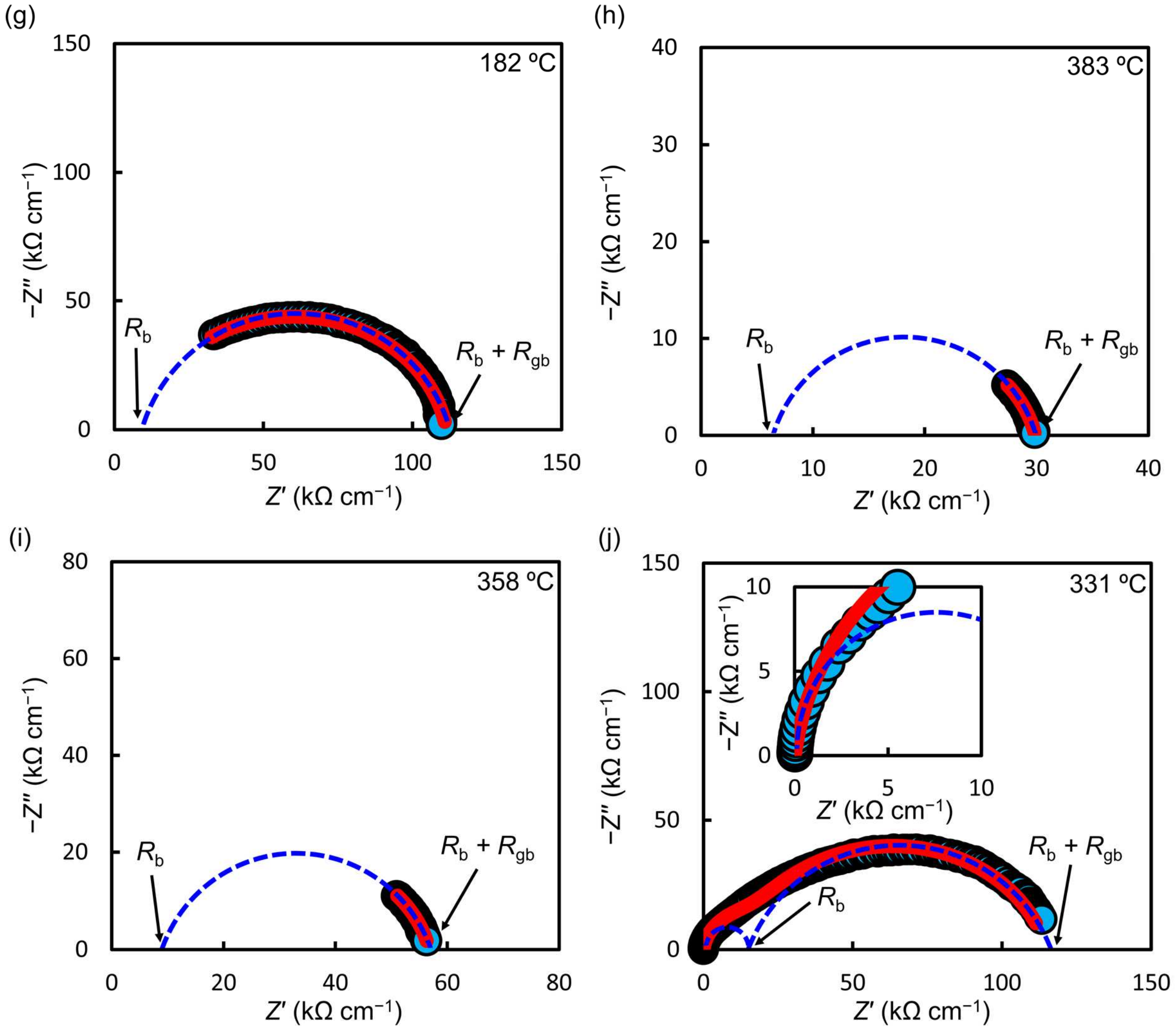

Figure S4. Complex impedance plane plots of Bi_2LuO_4Cl recorded at (a) 552 ºC, (b) 392 ºC and (c) 264 ºC. Complex impedance plane plots of $Bi_{1.9}Te_{0.1}LuO_{4.05}Cl$ at (d) 492 ºC, (e) 338 ºC, (f) 284 ºC and (g) 182 ºC. Complex impedance plane plots of $Bi_{1.8}Te_{0.2}LuO_{4.1}Cl$ at (h) 383 ºC, (i) 358 ºC, and (g) 331 ºC. The red line and light blue circles denote the fit to the data and experimental data, respectively. No electrode response was visible for all compositions. The blue dashed line is the fitting curve. R_b and R_{gb} denote the bulk resistance and grain-boundary resistance, respectively.

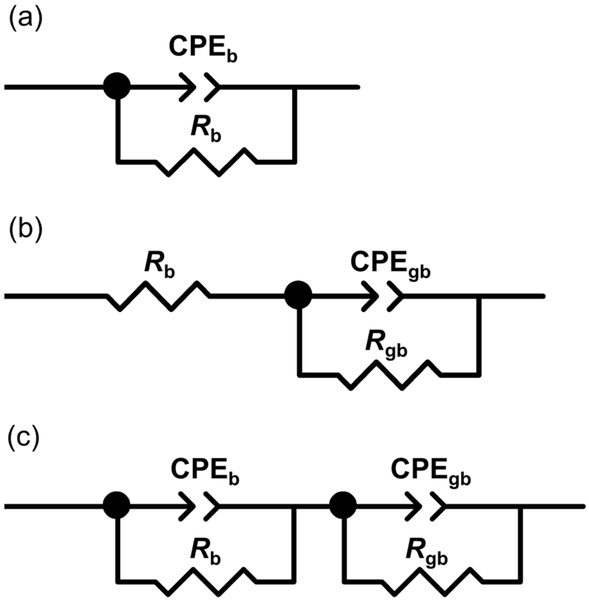

Figure S5. Equivalent circuits used to fit the impedance spectroscopy data. *R* and CPE denote a resistor and a constant phase element, respectively, where the subscripts b and gb stand for bulk and grain-boundary, respectively. See the details in Supplementary Note S1.

Supplementary Note S1.

The impedance data of Bi_2LuO_4Cl at 264–552 °C were analyzed using the equivalent circuit shown in Figure S5a. The bulk capacitance was ≈ 5×10^{-12} F cm^{-1}, indicating the bulk response.[1]

The impedance data of $Bi_{1.9}Te_{0.1}LuO_{4.05}Cl$ at 182–492 °C were analyzed using equivalent circuit shown in Figure S5b. The grain-boundary capacitance was ≈ 3×10^{-10} F cm^{-1}, indicating the grain-boundary response.[1]

The impedance data of $Bi_{1.8}Te_{0.2}LuO_{4.1}Cl$ at 358–383 °C were analyzed using equivalent circuit shown in Figure S5b. The grain-boundary capacitance was ≈ 1×10^{-10} F cm^{-1}, indicating the grain-boundary response.[1]

The impedance data of $Bi_{1.8}Te_{0.2}LuO_{4.1}Cl$ at 276–331 °C were analyzed using equivalent circuit shown in Figure S5c. The bulk and grain-boundary capacitances were ≈ 3×10^{-11} F cm^{-1} and ≈ 1×10^{-10} F cm^{-1}, indicating the bulk and grain-boundary responses, respectively.[1]

Supplementary Note S2.

We have investigated the oxide ion diffusion mechanisms in $Bi_{18}Lu_9O_{36}Cl_9$ [= $(Bi_2LuO_4Cl)_9$] and $Bi_{16}Te_2Lu_9O_{37}Cl_9$ [= $(Bi_{1.78}Te_{0.22}LuO_{4.11}Cl)_9$] using the static DFT calculations. We have considered both vacancy and interstitial processes to examine the energy barrier E_m for oxide ion migration. To investigate the energy profiles for vacancy and interstitialcy oxide-ion migration, the relaxation of atom positions with the convergence criterion of 0.01 eV Å^{-1} was performed by moving an O anion step-by-step (Figs. S6–S9).

[1] $Bi_{18}Lu_9O_{36}Cl_9$ [= $(Bi_2LuO_4Cl)_9$]:

In ideal undoped Bi_2LuO_4Cl without Te species, nor vacancies or interstitial oxide ions can exist, so that mobile species such as vacancies and interstitials must first be created before the oxide ion can migrate. In real Bi_2LuO_4Cl crystals, intrinsic defects (vacancy-interstitial pairs) might be thermally created with a formation energy E_f. Thus, the activation energy E_a in the case of intrinsic defects is given by the following Eq. (1).[2–4]

$$E_\mathrm{a} = E_\mathrm{m} + E_\mathrm{f}/2 \qquad (1)$$

We assume that the Frenkel defects are introduced according to the following defect reaction equation using Kröger-Vink notation:

$$\mathrm{O_{O1}} + v_\mathrm{O2} \rightarrow \mathrm{O''_{O2}} + v^{\bullet\bullet}_\mathrm{O1} \qquad (2)$$

where O_{O1}, v_{O2}, $\mathrm{O''_{O2}}$, and $v^{\bullet\bullet}_\mathrm{O1}$ represent an oxide ion at the lattice O1 site, a vacancy at the interstitial O2 site, an oxide ion at the interstitial O2 site, and a vacancy at the lattice O1 site, respectively. The formation energy of the Frenkel defect E_f was estimated to be 2.63 eV by DFT calculations. The E_m value for the oxide ion migration via the vacancy mechanism was estimated to be 0.24 eV (Figure S6), which was significantly lower than that via the interstitialcy mechanism (0.66 eV; Figure S7). Therefore, using Eq. (1), the activation energy E_a was calculated to be 1.56 eV, which is in excellent agreement with experimental E_a value 1.53(4) eV (Figure 1). Thus, the oxide ions in Bi_2LuO_4Cl migrate via the vacancy mechanism.

[2] $Bi_{16}Te_2Lu_9O_{37}Cl_9$ [= $(Bi_{1.78}Te_{0.22}LuO_{4.11}Cl)_9$]:

[2-1] Interstitialcy mechanism: $Bi_{16}Te_2Lu_9O_{37}Cl_9$ has an interstitial oxide ion, thus, we do not need to create the intrinsic defects (vacancy-interstitial pairs) in the calculations of the activation energy for the interstitialcy migration. The energy barriers E_m for oxide ion migration via the interstitialcy mechanism were estimated to be 0.62 eV (Figure S8). Thus, the activation energies for the interstitialcy oxide ion diffusion were also 0.62 eV.

[2-2] Vacancy mechanism: $Bi_{16}Te_2Lu_9O_{37}Cl_9$ has no oxygen vacancies, thus, the oxygen vacancies must first be created before the oxide ion can migrate via the vacancy mechanism. In real $Bi_{16}Te_2Lu_9O_{37}Cl_9$ crystals, intrinsic defects (vacancy-interstitial pairs) might be thermally created with a formation energy E_f. Thus, the activation energy E_a in the case of intrinsic defects is given by the Eq. (1). We assume that the

Frenkel defects are introduced according to the Eq. (2). The formation energy of the Frenkel defect E_f was calculated to be 2.36 eV. The E_m value for the oxide ion migration via the vacancy mechanism was estimated to be 0.18 eV (Figure S9). Therefore, using Eq. (1), the activation energy E_a was calculated to be 1.36 eV.

[2-3] Discussion: In $Bi_{16}Te_2Lu_9O_{37}Cl_9$, the activation energy for the interstitialcy mechanism E_a = 0.62 eV was lower than that for the vacancy mechanism E_a = 1.36 eV, strongly suggesting that the oxide-ion migration occurs via the interstitialcy mechanism, which is consistent with the AIMD simulations (Figure 6) and MEM NSLD distributions (Figure 5).

[3] Discussion:

It is interesting to point out that the oxide ion migration mechanism of Te-doped composition $Bi_{16}Te_2Lu_9O_{37}Cl_9$ [= $(Bi_{1.78}Te_{0.22}LuO_{4.11}Cl)_9$] is very different from that of undoped mother material $Bi_{18}Lu_9O_{36}Cl_9$ [= $(Bi_2LuO_4Cl)_9$]. Namely, the $Bi_{16}Te_2Lu_9O_{37}Cl_9$ exhibits the interstitialcy oxide ion diffusion, while the $Bi_{18}Lu_9O_{36}Cl_9$ shows the oxide ion diffusion via vacancy mechanism. The activation energy in $Bi_{16}Te_2Lu_9O_{37}Cl_9$ (E_a = 0.62 eV) is significantly lower than that in $Bi_{18}Lu_9O_{36}Cl_9$ (E_a = 1.56 eV), which is consistent with experimental activation energies. Therefore, the low activation energies in Te-doped compositions (0.577 and 0.61 eV) can be ascribed to the interstitialcy mechanism for oxide ion diffusion, leading to the high oxide ion conductivity at low temperatures.

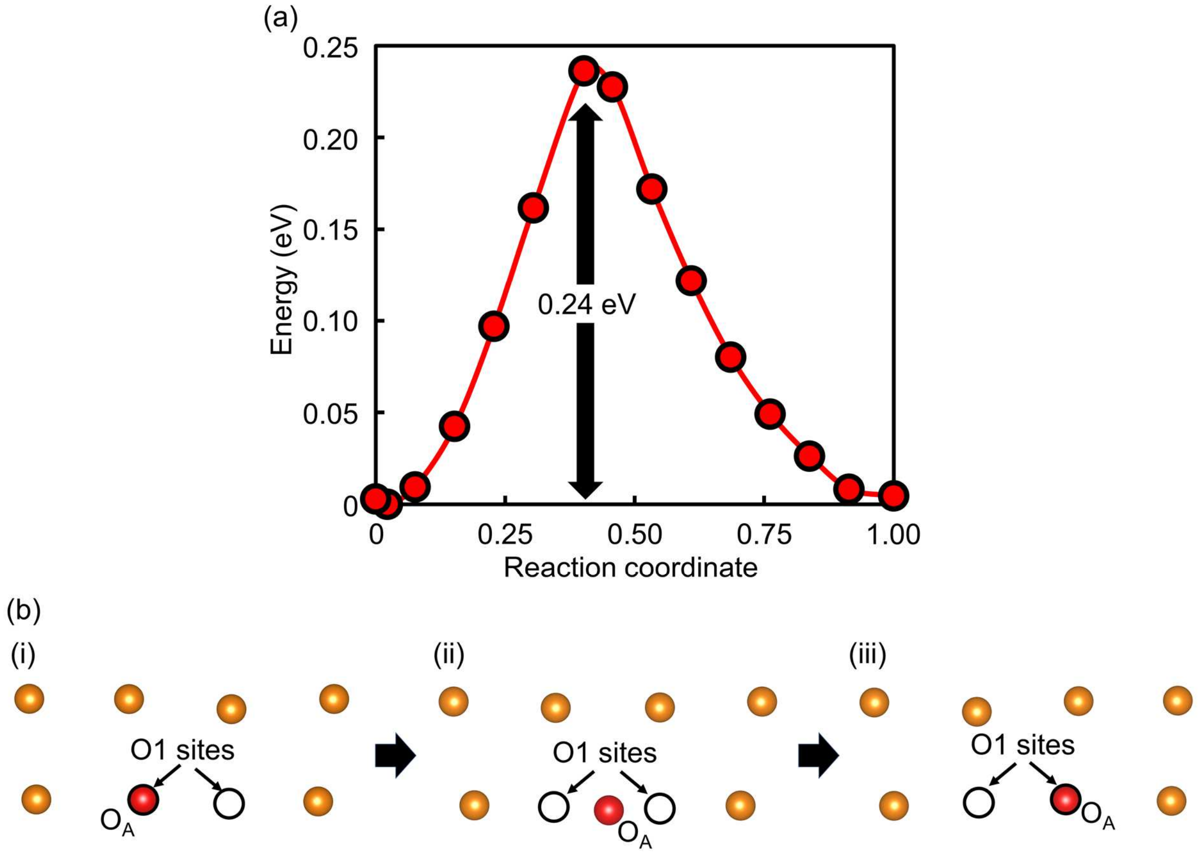

Figure S6 (a) Energy profile for the migration of the oxide ion via the vacancy mechanism in $Bi_{18}Lu_9O_{36}Cl_9$ [= $(Bi_2LuO_4Cl)_9$], which was investigated by static DFT calculations. In the panel (b), the figures (i), (ii), and (iii) show the initial, transition, and final states, respectively. Between the initial and final states, the red oxide ion O_A migrates from an O1 lattice site to a neighboring O1 site which is vacant at the initial state. Orange sphere represents an oxygen atom at the O1 site.

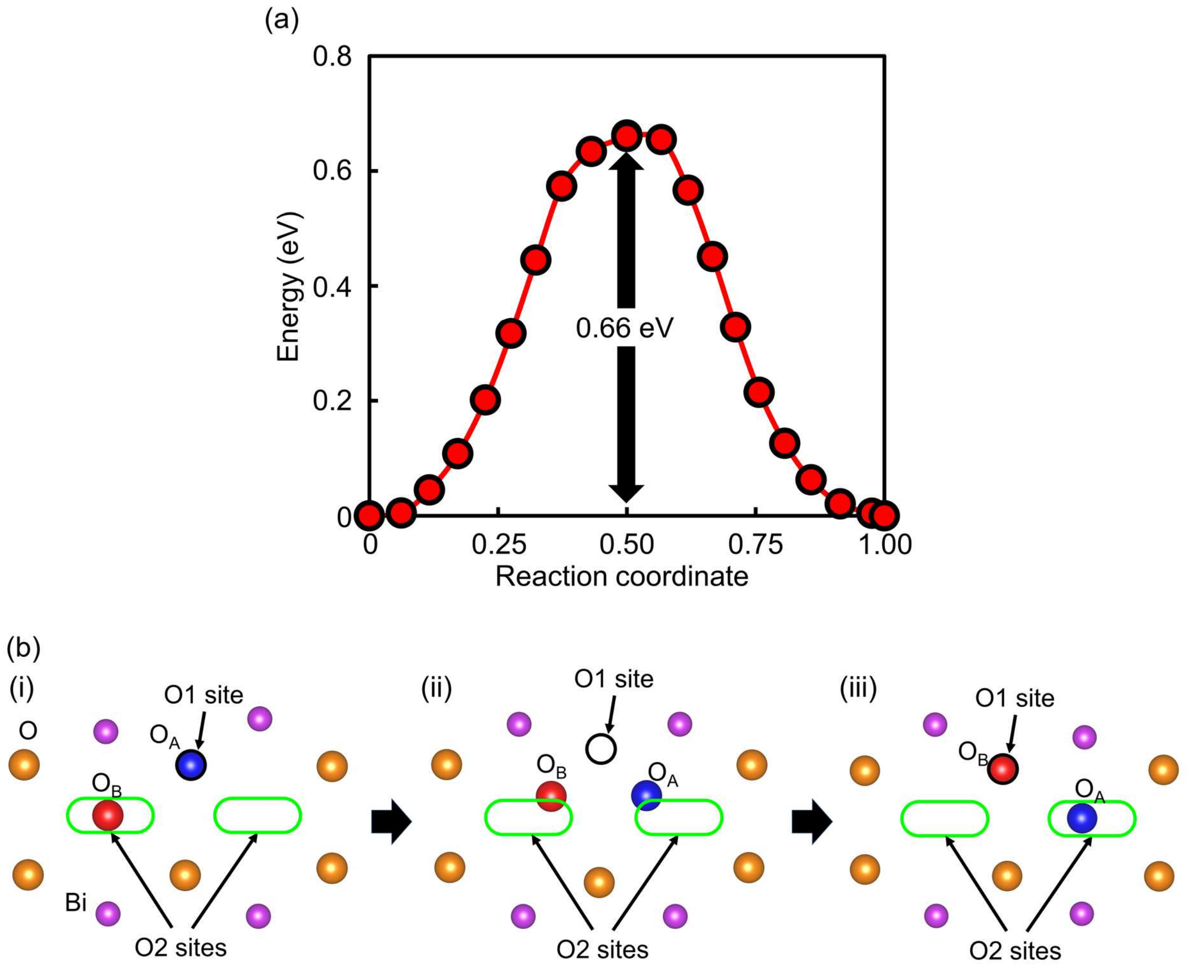

Figure S7 (a) Energy profile for the migration of the oxide ion via the interstitialcy mechanism in $Bi_{18}Lu_9O_{36}Cl_9$ [= $(Bi_2LuO_4Cl)_9$], which was investigated by static DFT calculations. In the panel (b), the figures (i), (ii), and (iii) show the initial, transition, and final states, respectively. Between the initial and final states, one red oxide ion O_B migrates from an O2 interstitial site to a neighboring O1 lattice site, pushing another blue oxide ion O_A from the O1 site to the neighboring vacant interstitial O2 site. Orange and purple spheres represent an oxygen atom at the O1 site and a Bi atom, respectively.

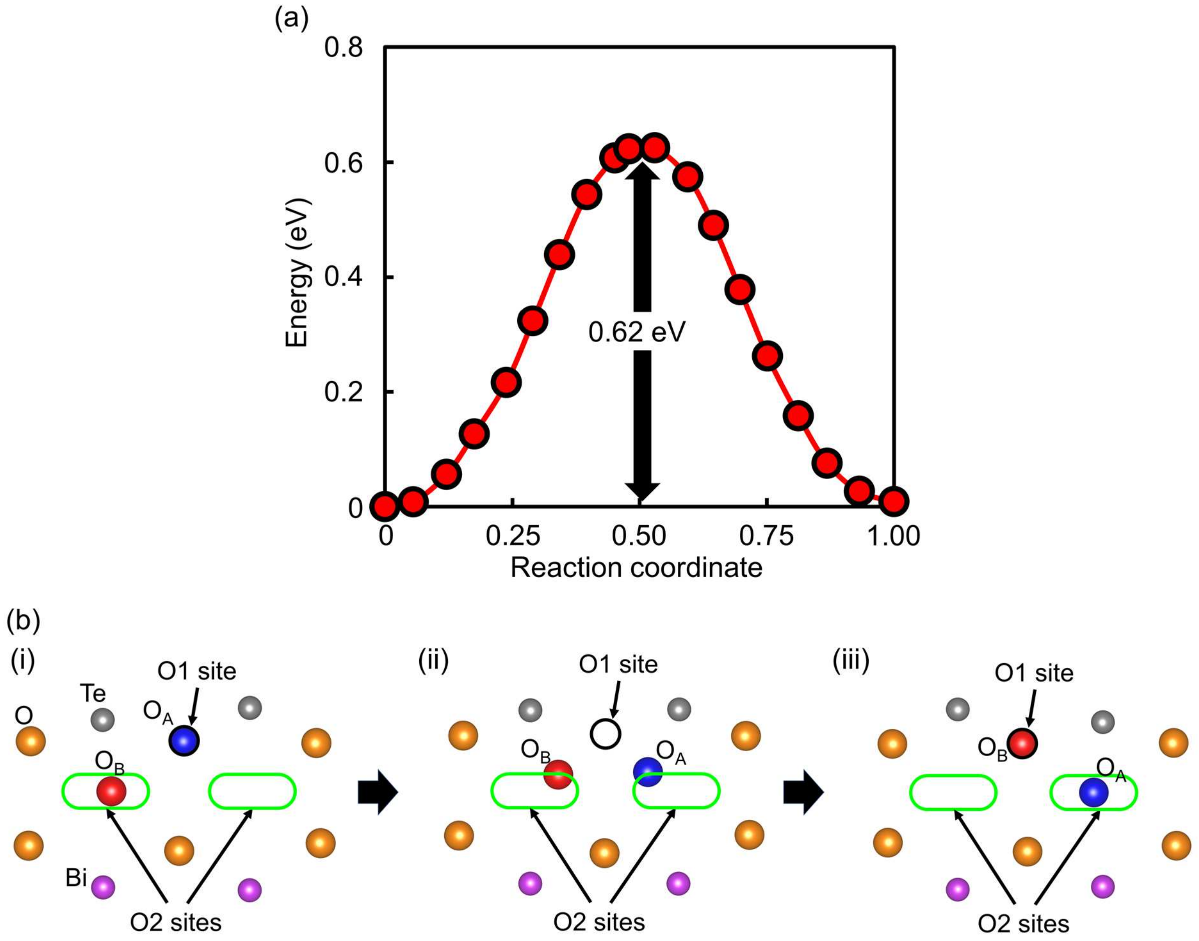

Figure S8 (a) Energy profile for the migrations of the oxide ion via the interstitialcy mechanism in $Bi_{16}Te_2Lu_9O_{37}Cl_9$ [= $(Bi_{1.78}Te_{0.22}LuO_{4.11}Cl)_9$], which was investigated by static DFT calculations. In the panel (b), the figures (i), (ii), and (iii) show the initial, transition, and final states, respectively. Between the initial and final states, one red oxide ion O_B migrates from an O2 interstitial site to a neighboring O1 lattice site, pushing another blue oxide ion O_A from the O1 site to the neighboring vacant interstitial O2 site. Orange, purple and gray spheres represent an oxygen atom at the O1 site, a Bi atom and a Te atom, respectively.

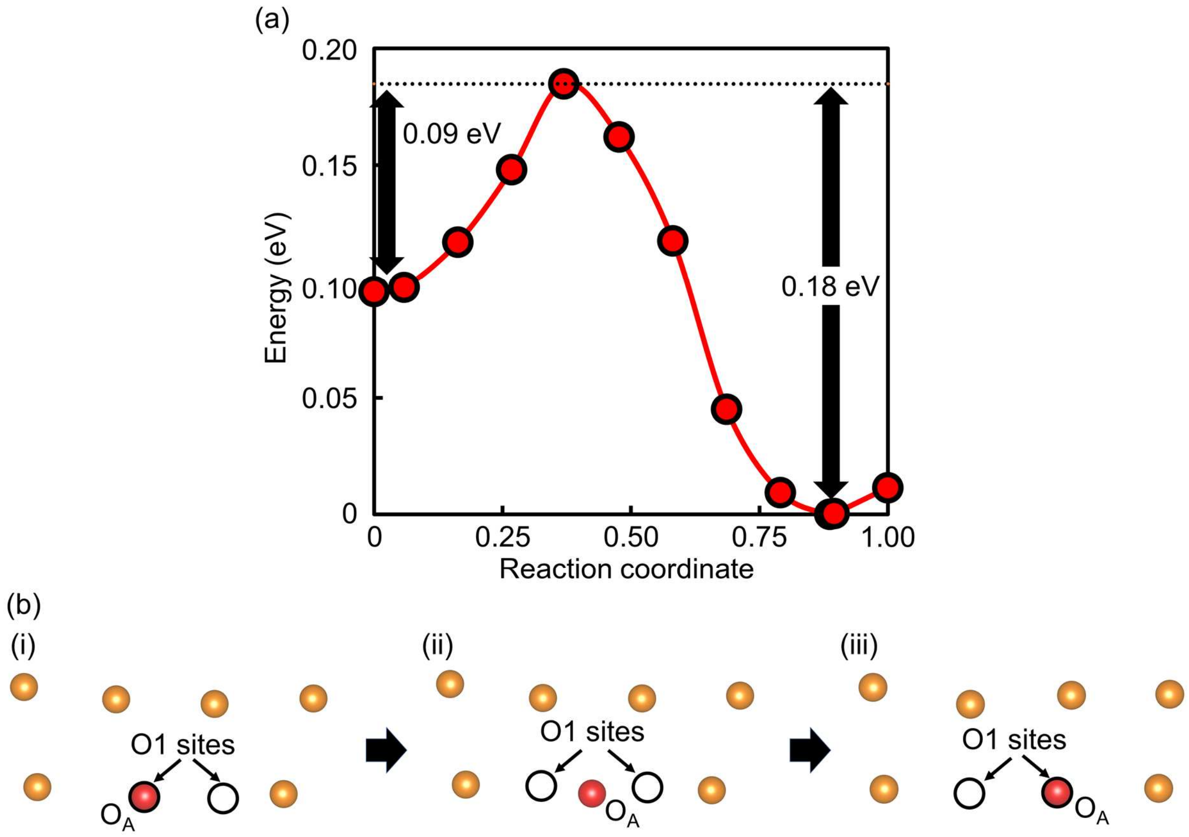

Figure S9 (a) Energy profile for the migrations for oxide ion via the vacancy mechanism in $Bi_{16}Te_2Lu_9O_{37}Cl_9$ [= $(Bi_{1.78}Te_{0.22}LuO_{4.11}Cl)_9$], which was investigated by static DFT calculations. In the panel (b), the figures (i), (ii), and (iii) show the initial, transition, and final states, respectively. Between the initial and final states, the red oxide ion O_A migrates from an O1 lattice site to a neighboring O1 site which is vacant at the initial state. Orange sphere represents an oxygen atom at the O1 site.

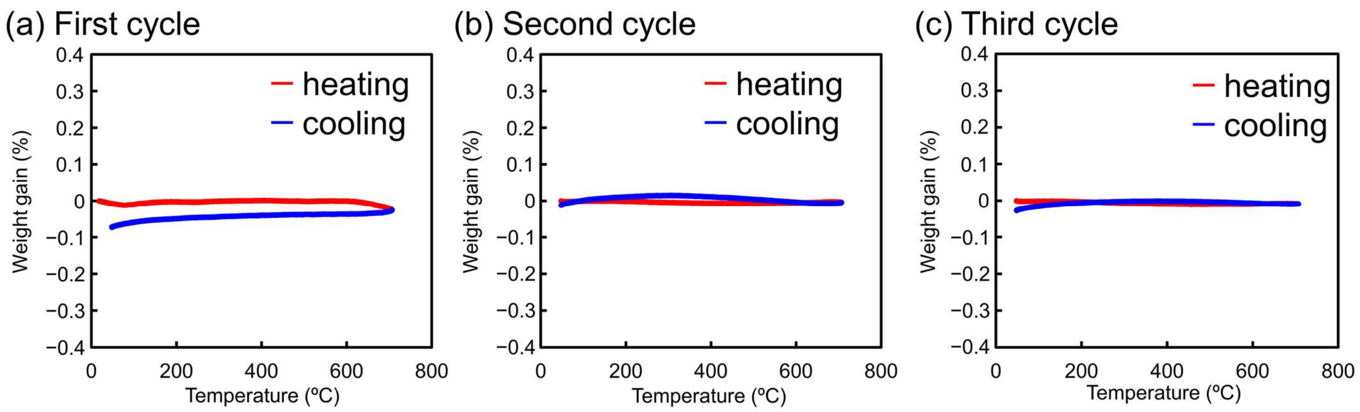

Figure S10. Results of the thermogravimetric (TG) analysis of $Bi_{1.9}Te_{0.1}LuO_{4.05}Cl$ in (a) first, (b) second and (b) third heating and cooling cycles under dry nitrogen flow.

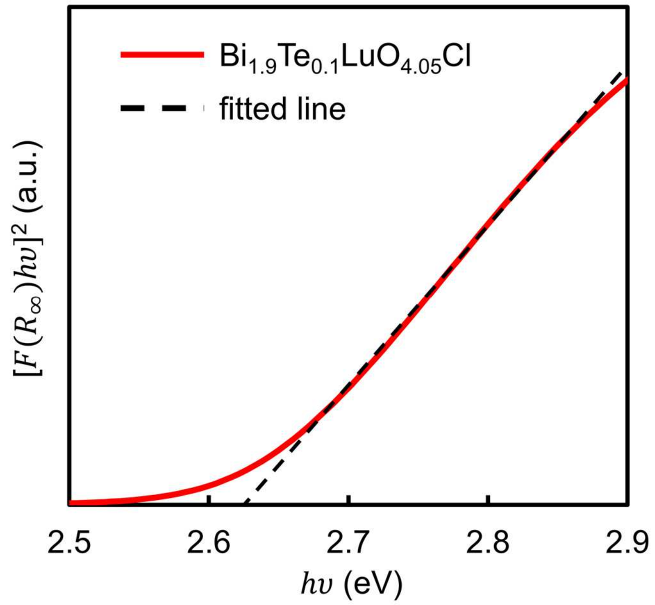

Figure S11. Tauc plot for the direct band gap of $Bi_{1.9}Te_{0.1}LuO_{4.05}Cl$. The band gap was estimated to be 2.62 eV.

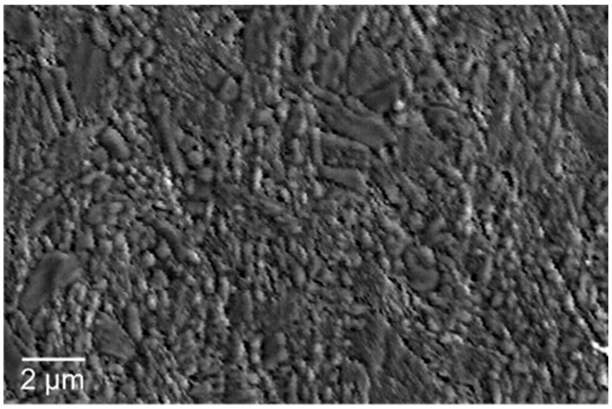

Figure S12. Scanning electron microscope (SEM) micrograph of $Bi_{1.9}Te_{0.1}LuO_{4.05}Cl$. The average grain size was estimated to be 0.39 µm.

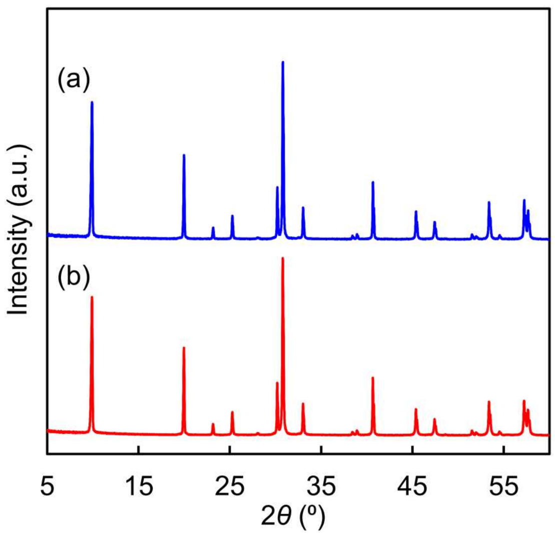

Figure S13. Cu Kα XRD patterns measured at room temperature of $Bi_{1.9}Te_{0.1}LuO_{4.05}Cl$ samples (a) before and (b) after the conductivity measurements at different $P(O_2)$ atmospheres. No degradation was observed in the XRD pattern (b).

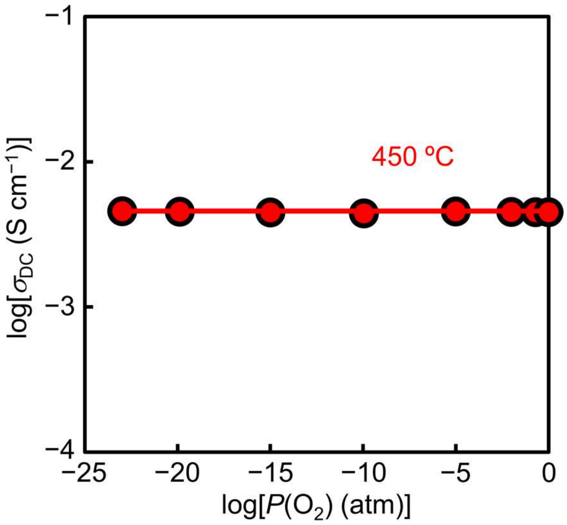

Figure S14. Oxygen partial pressure $P(O_2)$ dependence of the DC electrical conductivity σ_{DC} of a high-density $Bi_{1.9}Te_{0.1}LuO_{4.05}Cl$ pellet (relative density: 97%) at 450 ºC.

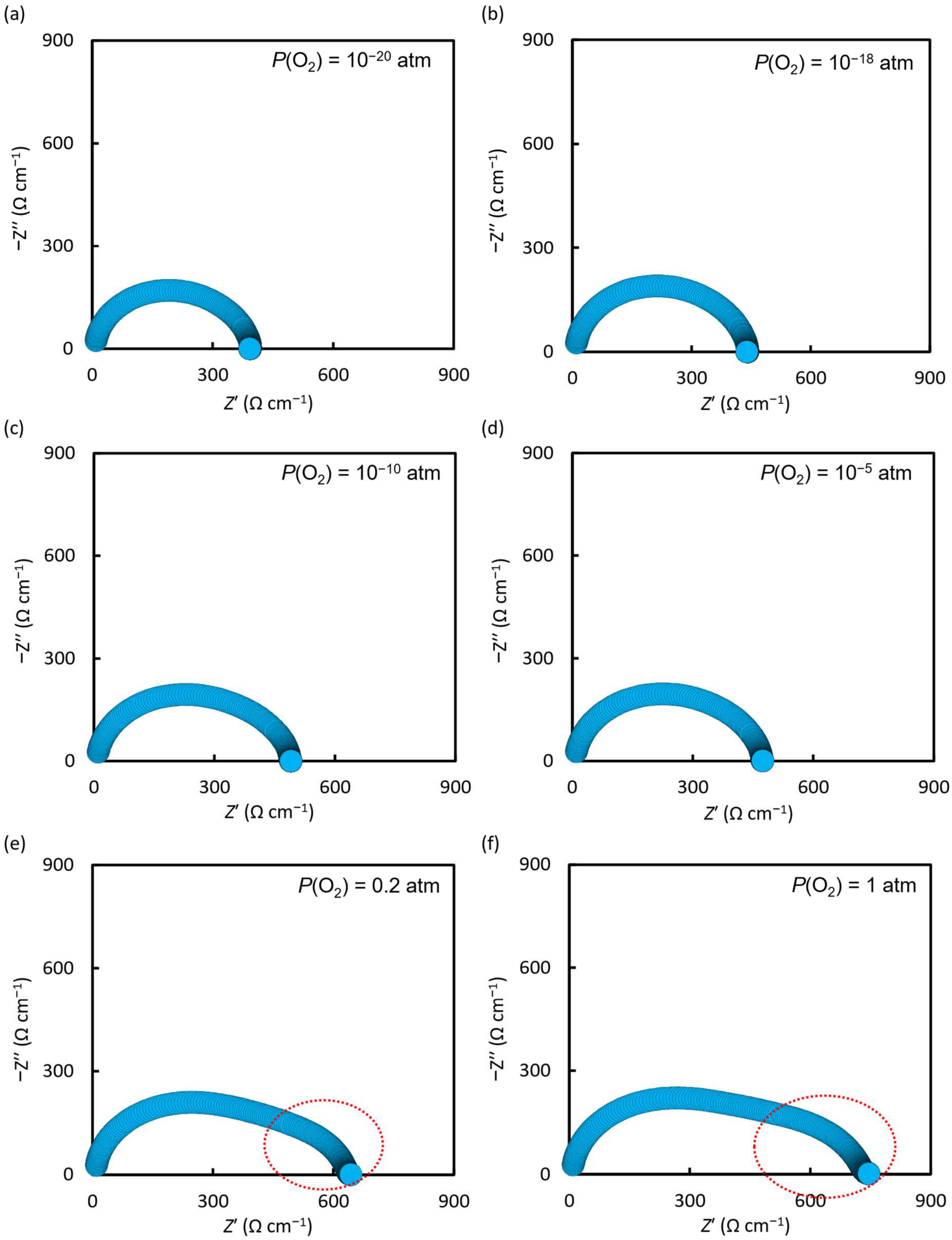

Figure S15. Complex impedance plane plots of $Bi_{1.9}Te_{0.1}LuO_{4.05}Cl$ at 440 °C at (a) $P(O_2)$ = 10^{-20} atm, (b) $P(O_2)$ =10^{-18} atm, (c) $P(O_2)$ =10^{-10} atm, (d) $P(O_2)$ =10^{-5} atm, (e) $P(O_2)$ = 0.2 atm, and (f) $P(O_2)$ =1 atm. Each red dotted circle represents an additional impedance response.

Supplementary Note S3.

The DC electrical conductivity σ_{DC} values of a low-density $Bi_{1.9}Te_{0.1}LuO_{4.05}Cl$ pellet (relative density: 78%) at 431 °C was approximately 3×10^{-5} S cm^{-1} independent of the oxygen partial pressure $P(O_2)$ between 1.3×10^{-4} and 1.2×10^{-18} atm (Figure 2a) indicating the electrolyte domain. The σ_{DC} values below $P(O_2) = 10^{-20}$ atm were significantly higher than those at $P(O_2) = 1.3\times10^{-4}$ and 1.2×10^{-18} atm, which can be attributed to the contribution of electronic conduction due to the partial reduction of $Bi_{1.9}Te_{0.1}LuO_{4.05}Cl$. The σ_{DC} values at $P(O_2) = 0.2$ and 1 atm were slightly lower than σ_{DC} at $P(O_2) = 1.3\times10^{-4}$ atm, which could be due to partial oxidation.

The DC electrical conductivity measured by 4-proble method σ_{DC} of a $Bi_{1.9}Te_{0.1}LuO_{4.05}Cl$ pellet (relative density: 97%) at 450 °C was approximately 4.5×10^{-3} S cm^{-1} independent of oxygen partial pressure $P(O_2)$ in the $P(O_2)$ range between 1.1×10^{-23} and 1.0 atm (Figure S14). Therefore, the reduction of conductivity at $P(O_2)$ = 0.2 and 1 atm can be suppressed by using high-density $Bi_{1.9}Te_{0.1}LuO_{4.05}Cl$ pellet.

Similar results were obtained also in the total AC electrical conductivity values estimated using the impedance data σ_{total}^{AC}. At 440 °C, the σ_{total}^{AC} values of $Bi_{1.9}Te_{0.1}LuO_{4.05}Cl$ were 2×10^{-3} S cm^{-1} independent of oxygen partial pressure $P(O_2)$ in the $P(O_2)$ range between 10^{-5} and 10^{-18} atm (Figure S15b-d), indicating the electrolyte domain. The σ_{total}^{AC} at $P(O_2) = 10^{-20}$ atm was higher than those at $P(O_2) = 10^{-18}$ and 10^{-5} atm, which could be due to the contribution of electronic conduction at $P(O_2) = 10^{-20}$ atm (Figure S15a). The σ_{total}^{AC} at $P(O_2) = 0.2$ and 1 atm were lower than those at $P(O_2) = 10^{-18}$ and 10^{-5} atm, which could be due to partial oxidation (Figure S15e, f).

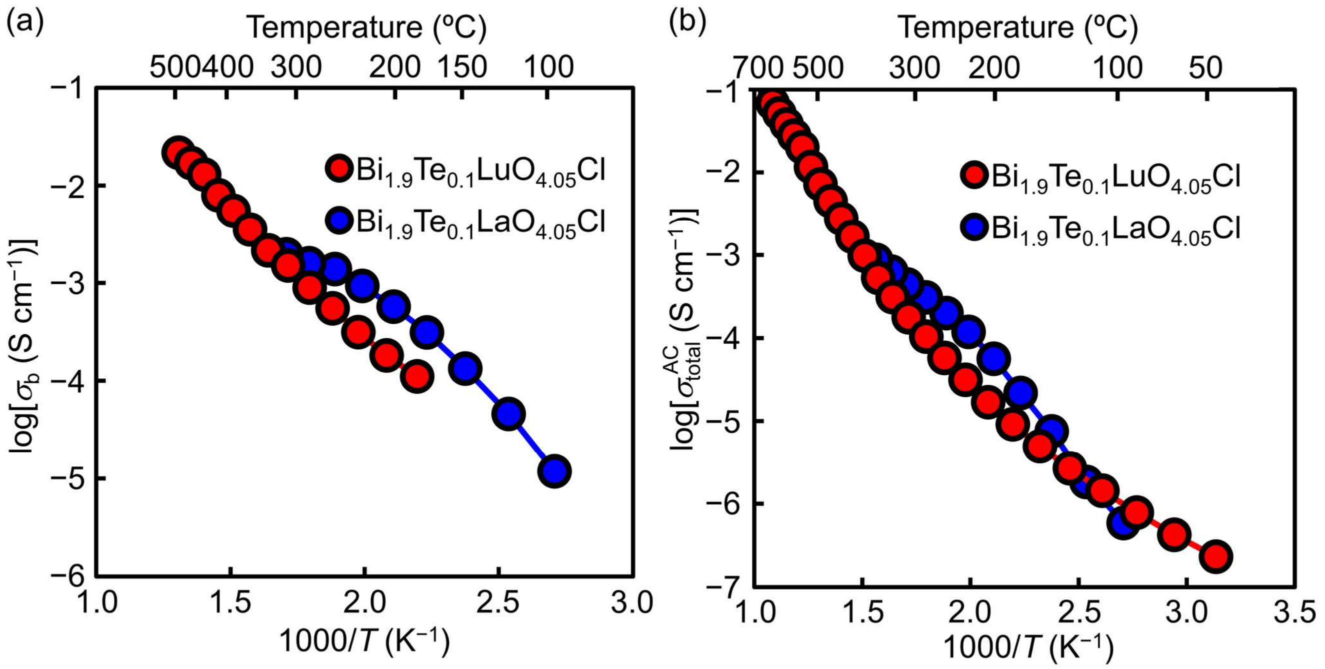

Figure S16. Arrhenius plots of (a) bulk conductivity σ_b and (b) total AC electrical conductivity σ_{total}^{AC} in dry nitrogen of $Bi_{1.9}Te_{0.1}LuO_{4.05}Cl$ and $Bi_{1.9}Te_{0.1}LaO_{4.05}Cl$. σ_{total}^{AC} was estimated using the total AC resistivity value ($R_b + R_{gb}$) extracted from the intercept of the grain-boundary semicircular arc (Figures S4c and S4d).

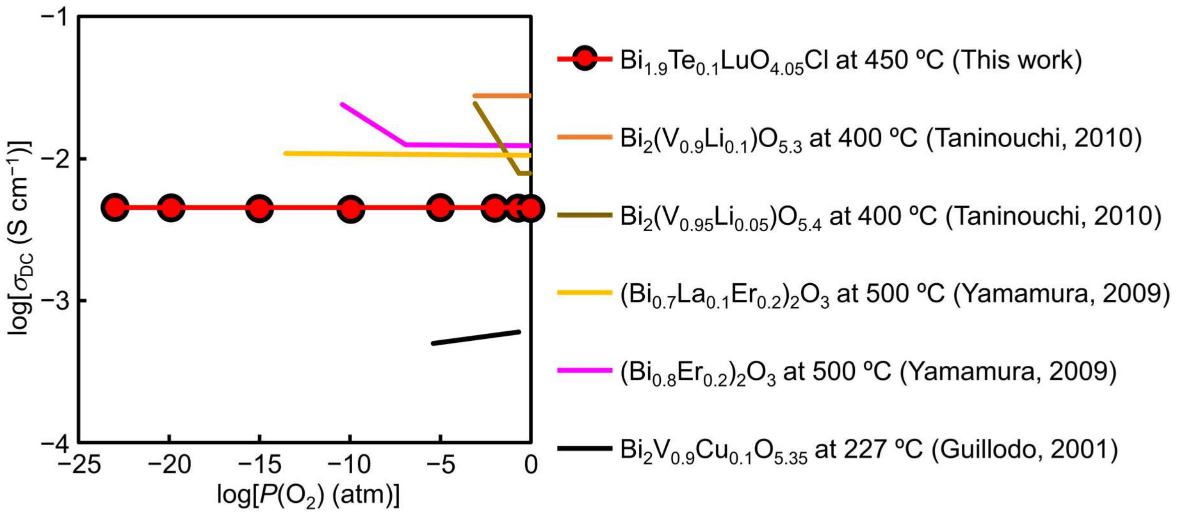

Figure S17. Oxygen partial pressure $P(O_2)$ dependence of the DC electrical conductivity, σ_{DC} of $Bi_{1.9}Te_{0.1}LuO_{4.05}Cl$, comparing with $Bi_2(V_{0.9}Li_{0.1})O_{5.3}$,[5] $Bi_2(V_{0.95}Li_{0.05})O_{5.4}$,[5] $(Bi_{0.7}La_{0.1}Er_{0.2})_2O_3$,[6] $(Bi_{0.8}Er_{0.2})_2O_3$,[6] and $Bi_2V_{0.9}Cu_{0.1}O_{5.35}$.[7] The σ_{DC} of $Bi_{1.9}Te_{0.1}LuO_{4.05}Cl$ is almost independent of $P(O_2)$ in the wide $P(O_2)$ range, and the electrolyte domain of $Bi_{1.9}Te_{0.1}LuO_{4.05}Cl$ is wider than those of the others. Thus, $Bi_{1.9}Te_{0.1}LuO_{4.05}Cl$ exhibits higher chemical and electrical stability than the others.

Table S1. Refined crystallographic parameters and reliability factors in Rietveld analysis of neutron diffraction data of $Bi_{1.9}Te_{0.1}LuO_{4.05}Cl$ at 25 °C.[a]

Site *X* Atom label	Atom *Y*	$g(Y; X)$[b]	Wyckoff site	*x*	*y*	*z*	U_{iso} or U_{eq} (Å^2)[c]	BVS
Bi/Te	Bi	0.95	2*h*	1/2	1/2	0.72155(2)	0.00436(6)	3.26
	Te	0.05	2*h*					
Lu	Lu	1	1*a*	0	0	0	0.00371(8)	2.84
O1	O	0.9916(4)	4*i*	1/2	0	0.84728(2)	0.00549(6)	−2.12
O2	O	0.0209(4)	4*n*	1/2	0.2225(18)	0	0.023(4)	−1.77
Cl	Cl	1	1*b*	0	0	1/2	0.01136(7)	−0.77

Site *X* Atom label	Atom *Y*	U_{11} (Å^2)	U_{22} (Å^2)	U_{33} (Å^2)	U_{12} (Å^2)	U_{13} (Å^2)	U_{23} (Å^2)
Bi/Te	Bi<br>Te	0.00229(9)	0.00229(9)	0.00850(15)	0	0	0
Lu	Lu	0.00266(14)	0.00266(14)	0.0058(2)	0	0	0
O1	O	0.00339(14)	0.00785(14)	0.00523(12)	0	0	0
Cl	Cl	0.00716(11)	0.00716(11)	0.01976(17)	0	0	0

[a] Crystal system: tetragonal, space group: *P*4/*mmm*, lattice parameters: a = 3.825316(4) Å and c = 8.856972(18) Å, R_{wp} = 0.0222, R_B = 0.0221, and R_F = 0.0213.
[b] $g(Y; X)$: Occupancy factor of atom *Y* at the *X* site. In a preliminary analysis, the occupancy factor of Bi/Te atom at the Bi/Te site $g(Bi_{0.95}Te_{0.05}$; Bi/Te) equaled to unity within three estimated standard deviation, thus, the $g(Bi_{0.95}Te_{0.05}$; Bi/Te) was fixed to unity in the final refinement. In another preliminary analysis, the occupancy factor of Lu atom at the Lu site *g*(Lu; Lu) was higher than unity, thus, the *g*(Lu; Lu) was fixed to unity in the final refinement. Linear constraint: *g*(O; O1) + *g*(O; O2) = 1.0125.
[c] U_{eq} denotes the equivalent and isotropic atomic displacement parameter and U_{iso} stands for the isotropic atomic displacement parameter.

Table S2. Refined crystallographic parameters and reliability factors in Rietveld analysis of neutron diffraction data of $Bi_{1.9}Te_{0.1}LuO_{4.05}Cl$ at 100 °C.[a]

Site *X* Atom label	Atom *Y*	$g(Y; X)$[b]	Wyckoff site	*x*	*y*	*z*	U_{iso} or U_{eq} (Å^2)[c]
Bi/Te	Bi	0.95	2*h*	1/2	1/2	0.72193(5)	0.00451(14)
	Te	0.05	2*h*				
Lu	Lu	1	1*a*	0	0	0	0.00407(19)
O1	O	0.9932(9)	4*i*	1/2	0	0.84736(5)	0.00630(14)
O2	O	0.0193(9)	4*n*	1/2	0.221(4)	0	0.011(8)
Cl	Cl	1	1*b*	0	0	1/2	0.01354(17)

Site *X* Atom label	Atom *Y*	U_{11} (Å^2)	U_{22} (Å^2)	U_{33} (Å^2)	U_{12} (Å^2)	U_{13} (Å^2)	U_{23} (Å^2)
Bi/Te	Bi<br>Te	0.0024(2)	0.0024(2)	0.0086(3)	0	0	0
Lu	Lu	0.0031(3)	0.0031(3)	0.0061(5)	0	0	0
O1	O	0.0042(3)	0.0086(3)	0.0061(3)	0	0	0
Cl	Cl	0.0092(3)	0.0092(3)	0.0223(4)	0	0	0

[a] Crystal system: tetragonal, space group: *P*4/*mmm*, lattice parameters: a = 3.828672(9) Å and c = 8.87003(4) Å, R_{wp} = 0.0225, R_B = 0.0217, and R_F = 0.0206.
[b] $g(Y; X)$: Occupancy factor of atom *Y* at the *X* site. Linear constraint: g(O; O1) + g(O; O2) = 1.0125.
[c] U_{eq} denotes the equivalent and isotropic atomic displacement parameter and U_{iso} stands for the isotropic atomic displacement parameter.

Table S3. Refined crystallographic parameters and reliability factors in Rietveld analysis of neutron diffraction data of $Bi_{1.9}Te_{0.1}LuO_{4.05}Cl$ at 200 °C.[a]

Site *X* Atom label	Atom *Y*	$g(Y; X)$[b]	Wyckoff site	*x*	*y*	*z*	U_{iso} or U_{eq} (Å^2)[c]
Bi/Te	Bi	0.95	2*h*	1/2	1/2	0.72219(5)	0.00622(15)
	Te	0.05	2*h*				
Lu	Lu	1	1*a*	0	0	0	0.0059(2)
O1	O	0.9942(10)	4*i*	1/2	0	0.84755(5)	0.00844(15)
O2	O	0.0183(10)	4*n*	1/2	0.223(5)	0	0.027(10)
Cl	Cl	1	1*b*	0	0	1/2	0.01830(19)

Site *X* Atom label	Atom *Y*	U_{11} (Å^2)	U_{22} (Å^2)	U_{33} (Å^2)	U_{12} (Å^2)	U_{13} (Å^2)	U_{23} (Å^2)
Bi/Te	Bi<br>Te	0.0043(2)	0.0043(2)	0.0100(4)	0	0	0
Lu	Lu	0.0042(3)	0.0042(3)	0.0092(5)	0	0	0
O1	O	0.0064(4)	0.0109(3)	0.0080(3)	0	0	0
Cl	Cl	0.0135(3)	0.0135(3)	0.0280(4)	0	0	0

[a] Crystal system: tetragonal, space group: *P*4/*mmm*, lattice parameters: a = 3.833894(10) Å and c = 8.88966(4) Å, R_{wp} = 0.0218, R_B = 0.0215, and R_F = 0.0206.

[b] $g(Y; X)$: Occupancy factor of atom *Y* at the *X* site. Linear constraint: g(O; O1) + g(O; O2) = 1.0125.

[c] U_{eq} denotes the equivalent and isotropic atomic displacement parameter and U_{iso} stands for the isotropic atomic displacement parameter.

Table S4. Refined crystallographic parameters and reliability factors in Rietveld analysis of neutron diffraction data of $Bi_{1.9}Te_{0.1}LuO_{4.05}Cl$ at 300 °C.[a]

Site X Atom label	Atom Y	$g(Y; X)$[b]	Wyckoff site	x	y	z	U_{iso} or U_{eq} (Å^2)[c]
Bi/Te	Bi	0.95	2h	1/2	1/2	0.72265(5)	0.00686(16)
	Te	0.05	2h				
Lu	Lu	1	1a	0	0	0	0.0064(2)
O1	O	0.9948(11)	4i	1/2	0	0.84777(5)	0.00901(16)
O2	O	0.0177(11)	4n	1/2	0.227(5)	0	0.021(11)
Cl	Cl	1	1b	0	0	1/2	0.0208(2)

Site X Atom label	Atom Y	U_{11} (Å^2)	U_{22} (Å^2)	U_{33} (Å^2)	U_{12} (Å^2)	U_{13} (Å^2)	U_{23} (Å^2)
Bi/Te	Bi<br>Te	0.0047(2)	0.0047(2)	0.0111(4)	0	0	0
Lu	Lu	0.0041(4)	0.0041(4)	0.0109(6)	0	0	0
O1	O	0.0066(4)	0.0120(4)	0.0084(3)	0	0	0
Cl	Cl	0.0158(3)	0.0158(3)	0.0308(5)	0	0	0

[a] Crystal system: tetragonal, space group: $P4/mmm$, lattice parameters: a = 3.839512(10) Å and c = 8.91007(4) Å, R_{wp} = 0.0219, R_B = 0.0223, and R_F = 0.0217.
[b] $g(Y; X)$: Occupancy factor of atom Y at the X site. Linear constraint: g(O; O1) + g(O; O2) = 1.0125.
[c] U_{eq} denotes the equivalent and isotropic atomic displacement parameter and U_{iso} stands for the isotropic atomic displacement parameter.

Table S5. Refined crystallographic parameters and reliability factors in Rietveld analysis of neutron diffraction data of $Bi_{1.9}Te_{0.1}LuO_{4.05}Cl$ at 400 °C.[a]

Site *X* Atom label	Atom *Y*	$g(Y; X)$[b]	Wyckoff site	*x*	*y*	*z*	U_{iso} or U_{eq} (Å^2)[c]
Bi/Te	Bi	0.95	2*h*	1/2	1/2	0.72290(5)	0.00881(18)
	Te	0.05	2*h*				
Lu	Lu	1	1*a*	0	0	0	0.0083(2)
O1	O	0.9962(11)	4*i*	1/2	0	0.84807(5)	0.01268(18)
O2	O	0.0163(11)	4*n*	1/2	0.228(5)	0	0.013(11)
Cl	Cl	1	1*b*	0	0	1/2	0.0267(2)
**Site *X* Atom label**	**Atom *Y***	U_{11} (Å^2)	U_{22} (Å^2)	U_{33} (Å^2)	U_{12} (Å^2)	U_{13} (Å^2)	U_{23} (Å^2)
Bi/Te	Bi<br>Te	0.0070(3)	0.0070(3)	0.0125(4)	0	0	0
Lu	Lu	0.0067(4)	0.0067(4)	0.0116(6)	0	0	0
O1	O	0.0103(4)	0.0148(4)	0.0130(4)	0	0	0
Cl	Cl	0.0217(3)	0.0217(3)	0.0367(5)	0	0	0

[a] Crystal system: tetragonal, space group: *P*4/*mmm*, lattice parameters: a = 3.845315(10) Å and c = 8.93106(4) Å, R_{wp} = 0.0209, R_B = 0.0230, and R_F = 0.0244.

[b] $g(Y; X)$: Occupancy factor of atom *Y* at the *X* site. Linear constraint: g(O; O1) + g(O; O2) = 1.0125.

[c] U_{eq} denotes the equivalent and isotropic atomic displacement parameter and U_{iso} stands for the isotropic atomic displacement parameter.

Table S6. Refined crystallographic parameters and reliability factors in Rietveld analysis of neutron diffraction data of $Bi_{1.9}Te_{0.1}LuO_{4.05}Cl$ at 500 °C.[a]

Site *X* Atom label	Atom *Y*	$g(Y; X)$[b]	Wyckoff site	*x*	*y*	*z*	U_{iso} or U_{eq} (Å^2)[c]
Bi/Te	Bi	0.95	2*h*	1/2	1/2	0.72325(6)	0.0113(2)
	Te	0.05	2*h*				
Lu	Lu	1	1*a*	0	0	0	0.0103(3)
O1	O	0.9969(12)	4*i*	1/2	0	0.84824(6)	0.0160(2)
O2	O	0.0156(12)	4*n*	1/2	0.211(6)	0	0.011(12)
Cl	Cl	1	1*b*	0	0	1/2	0.0323(3)
**Site *X* Atom label**	**Atom *Y***	U_{11} (Å^2)	U_{22} (Å^2)	U_{33} (Å^2)	U_{12} (Å^2)	U_{13} (Å^2)	U_{23} (Å^2)
Bi/Te	Bi, Te	0.0092(3)	0.0092(3)	0.0154(4)	0	0	0
Lu	Lu	0.0085(4)	0.0085(4)	0.0140(6)	0	0	0
O1	O	0.0142(5)	0.0177(5)	0.0161(4)	0	0	0
Cl	Cl	0.0264(4)	0.0264(4)	0.0440(6)	0	0	0

[a] Crystal system: tetragonal, space group: *P*4/*mmm*, lattice parameters: a = 3.851228(11) Å and c = 8.95288(4) Å, R_{wp} = 0.0203, R_B = 0.0225, and R_F = 0.0245.
[b] $g(Y; X)$: Occupancy factor of atom *Y* at the *X* site. Linear constraint: g(O; O1) + g(O; O2) = 1.0125.
[c] U_{eq} denotes the equivalent and isotropic atomic displacement parameter and U_{iso} stands for the isotropic atomic displacement parameter.

Table S7. Refined crystallographic parameters and reliability factors in Rietveld analysis of neutron diffraction data of $Bi_{1.9}Te_{0.1}LuO_{4.05}Cl$ at 600 °C.[a]

Site *X* Atom label	Atom *Y*	$g(Y; X)$[b]	Wyckoff site	*x*	*y*	*z*	U_{iso} or U_{eq} ($Å^2$)[c]
Bi/Te	Bi	0.95	2*h*	1/2	1/2	0.72355(6)	0.0132(2)
	Te	0.05	2*h*				
Lu	Lu	1	1*a*	0	0	0	0.0124(3)
O1	O	0.9969(12)	4*i*	1/2	0	0.84856(6)	0.0196(2)
O2	O	0.0156(12)	4*n*	1/2	0.228(7)	0	0.008(14)
Cl	Cl	1	1*b*	0	0	1/2	0.0387(3)
**Site *X* Atom label**	**Atom *Y***	**U_{11} ($Å^2$)**	**U_{22} ($Å^2$)**	**U_{33} ($Å^2$)**	**U_{12} ($Å^2$)**	**U_{13} ($Å^2$)**	**U_{23} ($Å^2$)**
Bi/Te	Bi, Te	0.0106(3)	0.0106(3)	0.0182(5)	0	0	0
Lu	Lu	0.0103(4)	0.0103(4)	0.0167(7)	0	0	0
O1	O	0.0181(5)	0.0206(5)	0.0201(4)	0	0	0
Cl	Cl	0.0330(4)	0.0330(4)	0.0499(6)	0	0	0

[a] Crystal system: tetragonal, space group: *P*4/*mmm*, lattice parameters: a = 3.857519(11) Å and c = 8.97434(5) Å, R_{wp} = 0.0191, R_B = 0.0229, and R_F = 0.0283.

[b] $g(Y; X)$: Occupancy factor of atom *Y* at the *X* site. Linear constraint: g(O; O1) + g(O; O2) = 1.0125.

[c] U_{eq} denotes the equivalent and isotropic atomic displacement parameter and U_{iso} stands for the isotropic atomic displacement parameter.

Table S8. Refined crystallographic parameters and reliability factors in Rietveld analysis of neutron diffraction data of $Bi_{1.9}Te_{0.1}LuO_{4.05}Cl$ at 700 °C.[a]

Site *X* Atom label	Atom *Y*	$g(Y; X)$[b]	Wyckoff site	*x*	*y*	*z*	U_{iso} or U_{eq} (Å^2)[c]
Bi/Te	Bi	0.95	2*h*	1/2	1/2	0.72381(5)	0.01599(19)
	Te	0.05	2*h*				
Lu	Lu	1	1*a*	0	0	0	0.0140(3)
O1	O	1	4*i*	1/2	0	0.84886(6)	0.02302(15)
O2	O	0.0125	4*n*	1/2	0.185(8)	0	0.011(11)
Cl	Cl	1	1*b*	0	0	1/2	0.0448(3)

Site *X* Atom label	Atom *Y*	U_{11} (Å^2)	U_{22} (Å^2)	U_{33} (Å^2)	U_{12} (Å^2)	U_{13} (Å^2)	U_{23} (Å^2)
Bi/Te	Bi<br>Te	0.0138(3)	0.0138(3)	0.0204(5)	0	0	0
Lu	Lu	0.0119(4)	0.0119(4)	0.0183(7)	0	0	0
O1	O	0.0222(5)	0.0244(5)	0.0224(4)	0	0	0
Cl	Cl	0.0390(4)	0.0390(4)	0.0563(6)	0	0	0

[a] Crystal system: tetragonal, space group: *P*4/*mmm*, lattice parameters: a = 3.864003(11)Å and c = 8.99679(4) Å, R_{wp} = 0.0183, R_B = 0.0246, and R_F = 0.0337.
[b] $g(Y; X)$: Occupancy factor of atom *Y* at the *X* site. In a preliminary analysis, the occupancy factor of Bi/Te atom at the Bi/Te site $g(Bi_{0.95}Te_{0.05}$; Bi/Te) equaled to unity within three estimated standard deviation, thus, the $g(Bi_{0.95}Te_{0.05}$; Bi/Te) was fixed to unity in the final refinement. In another preliminary analysis, the occupancy factor of Lu atom at the Lu site *g*(Lu; Lu) was higher than unity, thus, the *g*(Lu; Lu) was fixed to unity in the final refinement. In a preliminary analysis using a linear constraint: *g*(O; O1) + *g*(O; O2) = 1.0125, the occupancy factor of O atom at the O1 site *g*(O; O1) was higher than unity, thus, the *g*(O; O1) was fixed to unity in the final refinement.
[c] U_{eq} denotes the equivalent and isotropic atomic displacement parameter and U_{iso} stands for the isotropic atomic displacement parameter.

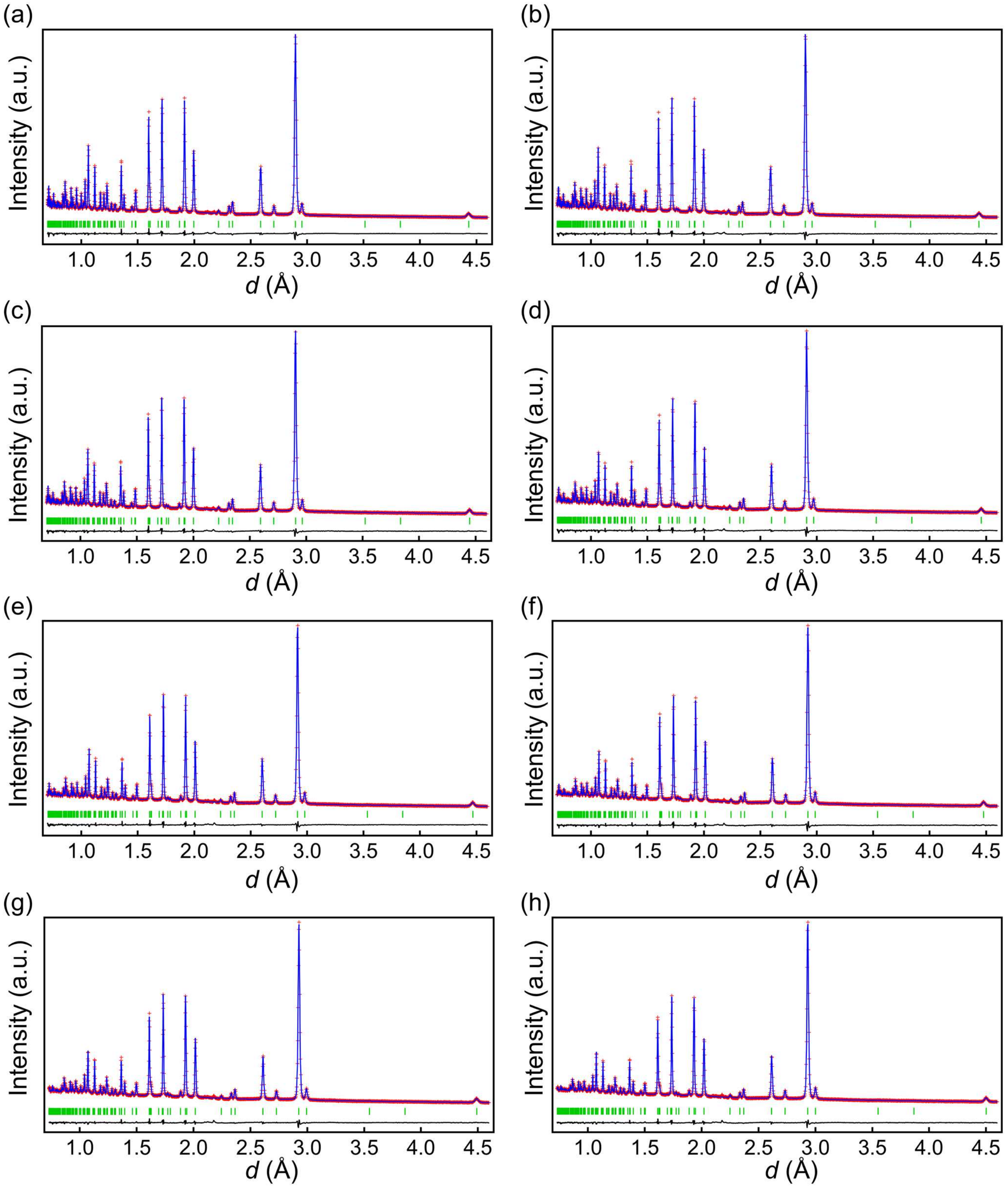

Figure S18. Rietveld patterns of $Bi_{1.9}Te_{0.1}LuO_{4.05}Cl$ for neutron powder diffraction data measured *in situ* at (a) 25 °C, (b) 100 °C, (c) 200 °C, (d) 300 °C, (e) 400 °C, (f) 500 °C, (g) 600 °C, and (h) 700 °C. Red crosses, blue solid line and black solid line stand for the experimental data, calculated intensities and difference pattern, respectively. Green vertical bars denote calculated Bragg peak positions of *P*4/*mmm* $Bi_{1.9}Te_{0.1}LuO_{4.05}Cl$.

Table S9. Refined crystallographic parameters and reliability factors in Rietveld analysis of neutron diffraction data of Bi_2LuO_4Cl at 25 °C.[a]

Site *X* Atom label	Atom *Y*	$g(Y; X)$[b]	Wyckoff site	x	y	z	U_{eq} (Å^2)[c]	U_{11} (Å^2)	U_{22} (Å^2)	U_{33} (Å^2)	BVS
Bi	Bi	1	2*h*	1/2	1/2	0.720884(16)	0.00872(3)	0.00684(5)	0.00684(5)	0.01248(8)	3.26
Lu	Lu	1	1*a*	0	0	0	0.00893(5)	0.00630(7)	0.00630(7)	0.01418(13)	2.75
O1	O	1	4*i*	1/2	0	0.847432(16)	0.01003(3)	0.00773(9)	0.00830(9)	0.01408(8)	−2.12
Cl	Cl	1	1*b*	0	0	1/2	0.01858(5)	0.01635(7)	0.01635(7)	0.02303(11)	−0.78

[a] Crystal system: tetragonal, space group: *P*4/*mmm*, lattice parameter a = 3.824172(2) Å and c = 8.869899(11) Å, R_{wp} = 0.0900, R_B = 0.0337 and R_F = 0.0381.
[b] $g(Y; X)$: Occupancy factor of atom *Y* at the *X* site. A preliminary Rietveld analysis was performed based on the 4*n* interstitial model where oxygen atoms were put at 4*i* lattice and 4*n* interstitial sites. In a preliminary analysis, the g(O; O2) was close to zero and the *R* factors were almost same as those for the model without interstitial oxygen atoms. Therefore, the model without interstitial oxygen atoms was used in the final refinement. In other preliminary analysis, the occupancy factor of Bi atom at the Bi site g(Bi; Bi) was higher than unity, thus, the g(Bi; Bi) was fixed to unity in the final refinement. In other preliminary analysis, the occupancy factor of Lu atom at the Lu site g(Lu; Lu) equaled to unity within three estimated standard deviation, thus, the g(Lu; Lu) was fixed to unity in the final refinement.
[c] Equivalent isotropic atomic displacement parameter (ADP). The anisotropic ADPs, U_{12}, U_{23}, and U_{31} are 0 for all the Bi, Lu, O, and Cl atoms.

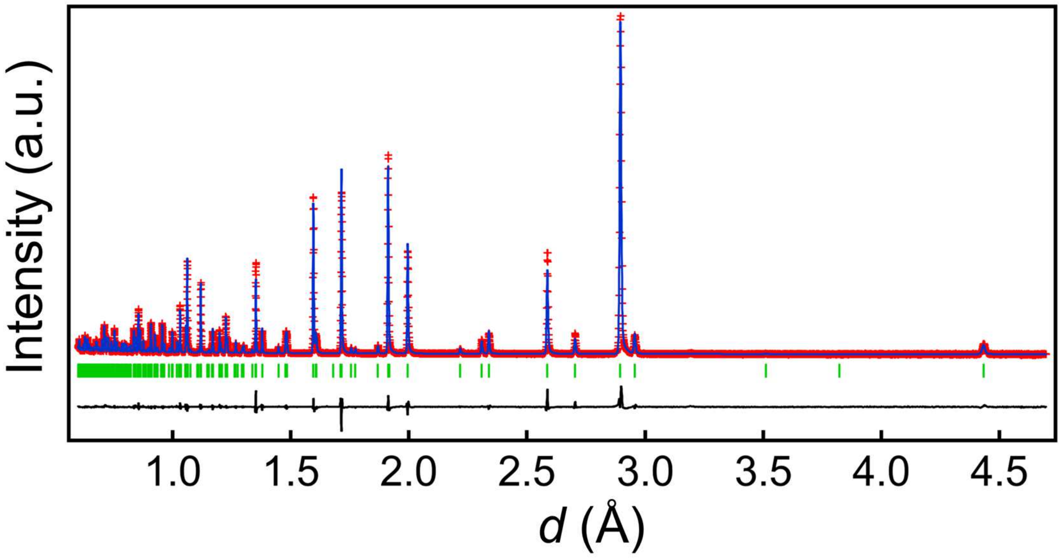

Figure S19. Rietveld pattern of Bi_2LuO_4Cl for neutron powder diffraction data measured at 25 °C. Red crosses, blue solid line and black solid line stand for the experimental data, calculated intensities and difference pattern, respectively. Green vertical bars denote calculated Bragg peak positions of *P*4/*mmm* Bi_2LuO_4Cl.

Supplementary Note S4. Crystal structure analysis using neutron diffraction of $Bi_{1.9}Te_{0.1}LuO_{4.05}Cl$

First, we analyzed the neutron diffraction data of $Bi_{1.9}Te_{0.1}LuO_{4.05}Cl$ taken at 400 °C using Rietveld method, maximum-entropy method (MEM), and structural model where the oxygen atoms are located only at the lattice 4*i* O1 site (named "model without interstitial oxygen atoms"). The neutron scattering length density (NSLD) distribution showed clear peaks at the Wyckoff 4*n* site (atomic coordinates: 1/2, ~0.22, 0) (dashed circles in Figures S20b,c and S21d). Therefore, we performed the structure analysis using the structural model where the oxygen atoms are located at both the interstitial 4*n* O2 and lattice 4*i* O1 sites (named "4*n* interstitial model") as shown in Figure 5a,b. The 4*n* interstitial model gave slightly lower reliability factors (R_{wp} = 2.09%, R_B = 2.30%, and R_F = 2.44%) compared to the model without interstitial oxygen atoms (R_{wp} = 2.11%, R_B = 2.50%, and R_F = 2.68%). Here, the R_{wp}, R_B, and R_F are the reliability (*R*) factors based on the weighted profile intensities, Bragg intensities, and structure factors, respectively. Yaguchi et al. used other interstitial model "1*c* interstitial model" in which the oxygen atoms are placed at both the interstitial 1*c* O2 and lattice 4*i* O1 sites.[8] The *R* factors for the 1*c* interstitial model (R_{wp} = 2.10%, R_B = 2.45%, R_F = 2.63%) were slightly higher than those for the 4*n* interstitial model. The MEM analysis of the 1*c* interstitial model indicated NSLD distributions around the 4*n* site (Figure S21f), which was inconsistent with the 1*c* interstitial model. The 4*n* interstitial and the 1*c* interstitial models gave bond-valence sums (BVSs) of −1.70 and −1.20 for O2 at 400 °C, respectively. BVS value for the 4*n* interstitial model −1.70 was closer to the formal charge of the oxide ion −2.0. In the 4*n* interstitial model, the occupancy factors at the O1 and O2 sites were refined to be 0.9962(11) and 0.0163(11), respectively. The presence of the interstitial oxide ion at the 4*n* site was also supported by the bond valence-based energy landscape for an oxide ion (Figure S22). All these results indicate the presence of interstitial oxygen atoms at the 4*n* site in the triple fluorite-like layer of $Bi_{1.9}Te_{0.1}LuO_{4.05}Cl$.

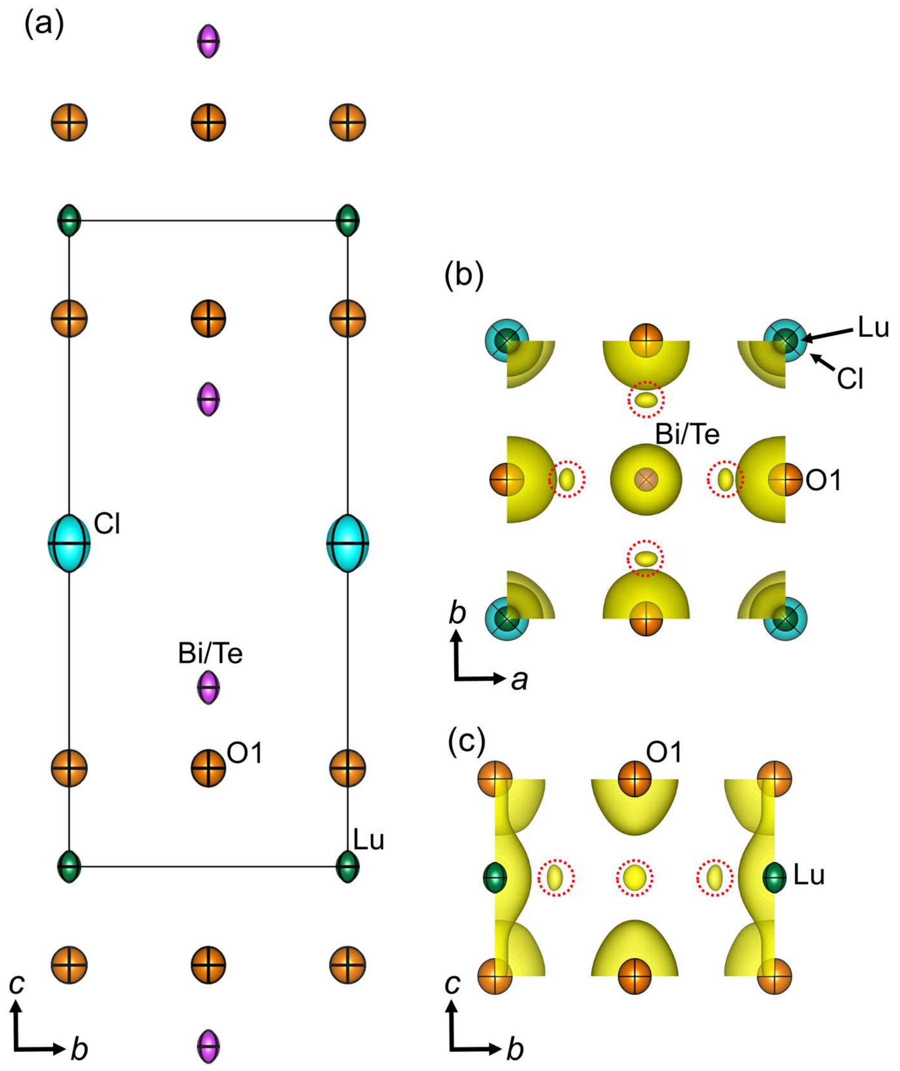

Figure S20. (a) Refined crystal structure and (b, c) corresponding yellow isosurfaces of maximum-entropy method (MEM) neutron scattering length densities (NSLDs) at 0.002 fm Å^{-3} in $Bi_{1.9}Te_{0.1}LuO_{4.05}Cl$, based on the model without interstitial oxygen atoms. Rietveld and MEM analyses were performed using the ND data of $Bi_{1.9}Te_{0.1}LuO_{4.05}Cl$ measured at 400 °C. Orange, light blue, purple and dark green spheres denote the O, Cl, Bi/Te and Lu atoms, respectively. Thermal ellipsoids are drawn at the 75% probability level. The black rectangle represents the unit cell. In panels b and c, the red dashed circles represent the isosurfaces of the NSLD at the 4*n* 0.5, 0.22, 0 sites, suggesting the interstitial O2 atoms at the 4*n* site.

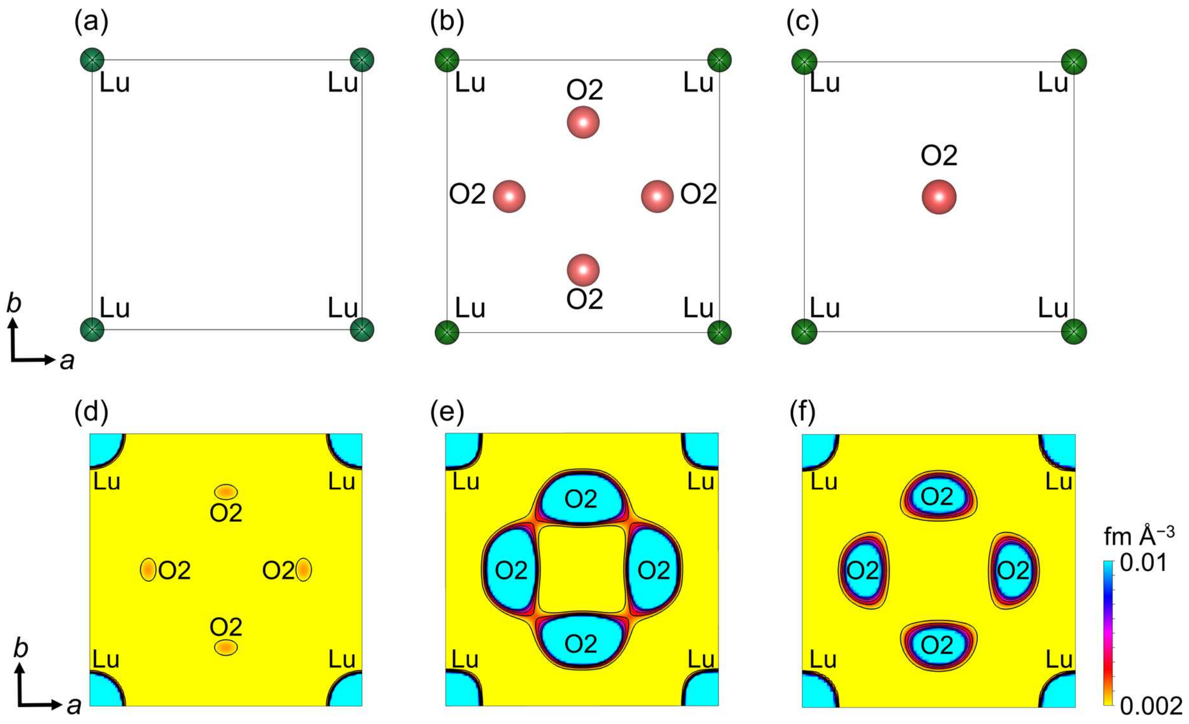

Figure S21. Refined crystal structures of $Bi_{1.9}Te_{0.1}LuO_{4.05}Cl$ based on (a) the model without interstitial oxygen atoms, (b) the 4*n* interstitial model, and (c) the 1*c* interstitial model viewed along the *c*-axis for $0.0 \leq x \leq 1.0$; $0 \leq y \leq 1.0$; $-0.1 \leq z \leq 0.1$ at 400 °C. MEM NSLD distributions of $Bi_{1.9}Te_{0.1}LuO_{4.05}Cl$ for (d) the model without interstitial oxygen atoms, (e) the 4*n* interstitial model, and (f) the 1*c* interstitial model on the *ab* plane ($0 \leq x \leq 1.0$; $0 \leq y \leq 1.0$; $z = 0$) at 400 °C. In (d), (e), and (f), the contour lines are from 0.002 to 0.01 fm $Å^{-3}$ with the step of 0.001 fm $Å^{-3}$. The refined struture for the model without interstitial oxygen atoms (a) is inconsistent with the MEM NSLD distribution for the model without interstitial oxygen atoms (d). The refined struture for the 1*c* interstitial model (c) is inconsistent with the MEM NSLD distribution for the 1*c* interstitial model (f). In contrast, the refined structure for the 4*n* interstitial model (e) is consistent with the MEM NSLD distribution for the 4*n* interstitial model, validating the 4*n* interstitial model.

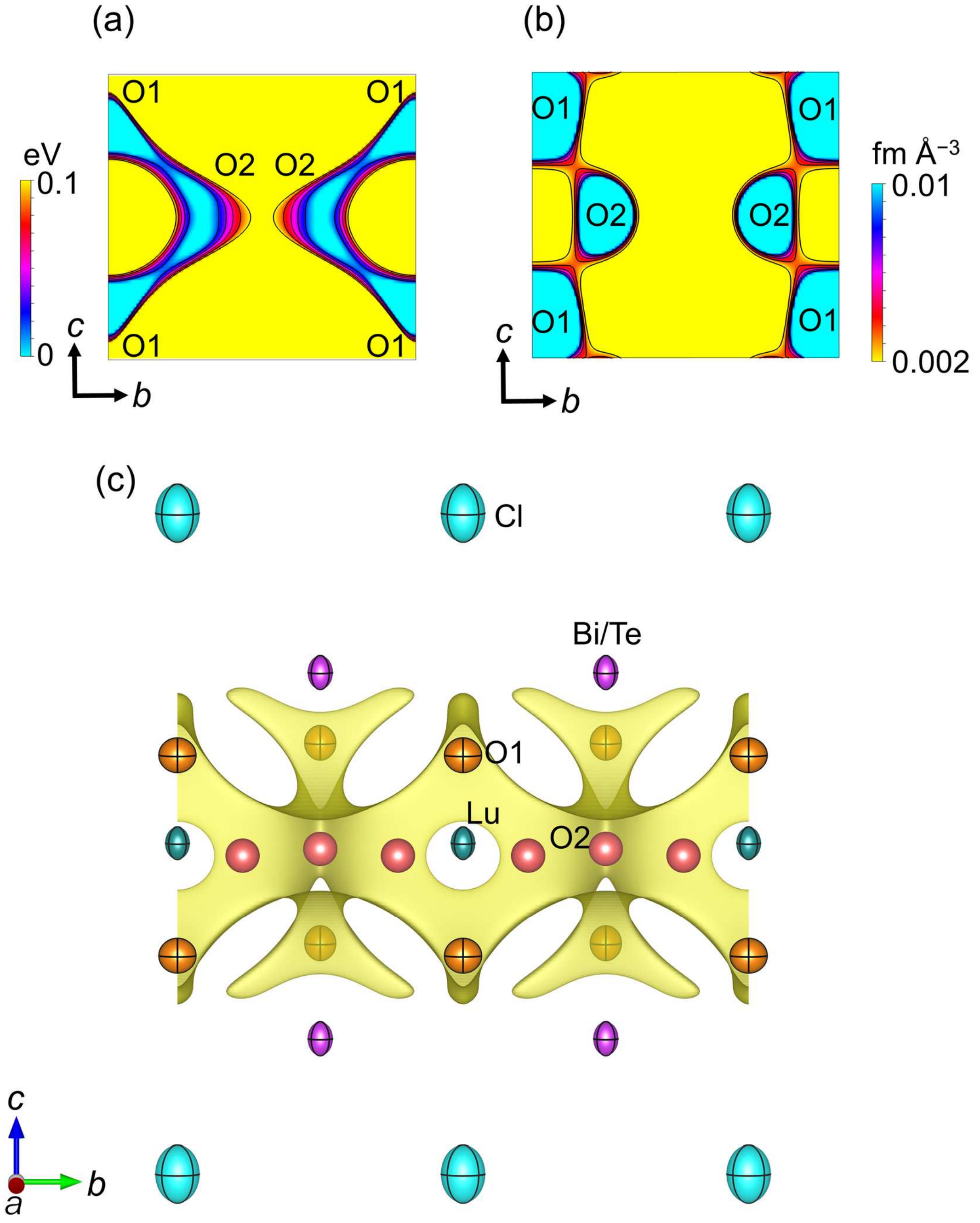

Figure S22. (a) Bond-valence-based energy (BVE) landscape of a test oxide ion and (b) MEM NSLD distribution at 400 °C on the *bc* plane of $Bi_{1.9}Te_{0.1}LuO_{4.05}Cl$ ($x = 0.5$; $0 \leq y \leq 1.0$; $-0.2 \leq z \leq 0.2$). Both BVE landscape and NSLD distribution are consistent with each other and show the oxide-ion diffusion pathways between the lattice O1 and interstitial O2 sites. Contour lines from −1.94 to −1.84 eV (0.02 eV step) in panel (a) where the BVE of a test oxide ion is set to 0 eV at the most stable position. Contour lines from 0.002 to 0.01 fm $Å^{-3}$ (0.0015 fm $Å^{-3}$ step) in panel (b). (c) BVE landscape of a test oxide ion with the yellow isosurfaces at 0.6 eV of $Bi_{1.9}Te_{0.1}LuO_{4.05}Cl$ at 400 °C ($0 \leq x \leq 0.5$; $0 \leq y \leq 2.0$; $-0.5 \leq z \leq 0.5$), showing the two-dimensional oxide ion diffusion in the triple fluorite-like layer.

Supplementary Note S5. Crystal structure analyses of synchrotron X-ray diffraction (SXRD) data for $Bi_{1.9}Te_{0.1}LuO_{4.05}Cl$

SXRD data of $Bi_{1.9}Te_{0.1}LuO_{4.05}Cl$ were successfully analyzed by the Rietveld method with the 4*n* interstitial model (Table S10 and Figure S23). The refined lattice parameter and atomic coordinates in the Rietveld analyses using SXRD data are consistent with those using neutron diffraction data (Table S1 and Table S10).

Table S10. Refined crystallographic parameters and reliability factors in Rietveld analysis of synchrotron XRD data of $Bi_{1.9}Te_{0.1}LuO_{4.05}Cl$ at 27 °C.[a]

Site *X* Atom label	Atom *Y*	$g(Y; X)$[b]	Wyckoff site	*x*	*y*	*z*	U_{iso} or U_{eq} (Å^2)[c]
Bi/Te	Bi	0.95	2*h*	1/2	1/2	0.720477(16)	0.00541(3)
	Te	0.05	2*h*				
Lu	Lu	1	1*a*	0	0	0	0.00402(5)
O1	O	0.9916	4*i*	1/2	0	0.84484(15)	0.0042(3)
O2	O	0.0209	4*n*	1/2	0.2225	0	= U_{iso}(O1)
Cl	Cl	1	1*b*	0	0	1/2	0.0151(3)
**Site *X* Atom label**	**Atom *Y***	**U_{11} (Å^2)**	**U_{22} (Å^2)**	**U_{33} (Å^2)**	**U_{12} (Å^2)**	**U_{13} (Å^2)**	**U_{23} (Å^2)**
Bi/Te	Bi, Te	0.00375(4)	0.00375(4)	0.00872(7)	0	0	0
Lu	Lu	0.00245(6)	0.00245(6)	0.00715(12)	0	0	0
Cl	Cl	0.0138(4)	0.0138(4)	0.0177(8)	0	0	0

[a] Crystal system: tetragonal, space group: *P*4/*mmm*, lattice parameters: a = 3.826108(3) Å and c = 8.855848(11) Å, R_{wp} = 0.0813, R_B = 0.0220, and R_F = 0.0206.
[b] Occupancy factor of atom *Y* at the *X* site. The *g*(O; O1) and *g*(O; O2) were fixed to the values refined using the ND data (Table S1). In a preliminary analysis, the occupancy factor of Lu atom at the Lu site *g*(Lu; Lu) was higher than unity, thus, the *g*(Lu; Lu) was fixed to unity in the final refinement. In another preliminary analysis, the occupancy factor of Cl atom at the Cl site *g*(Cl; Cl) equaled to unity within three estimated standard deviation, thus, the *g*(Cl; Cl) was fixed to unity in the final refinement. [c] U_{eq} denotes the equivalent and isotropic atomic displacement parameter and U_{iso} stands for the isotropic atomic displacement parameter. The occupancy factors of O1 and O2 atoms, and atomic coordinates of O2 atom were fixed to the values obtained using the neutron diffraction data.

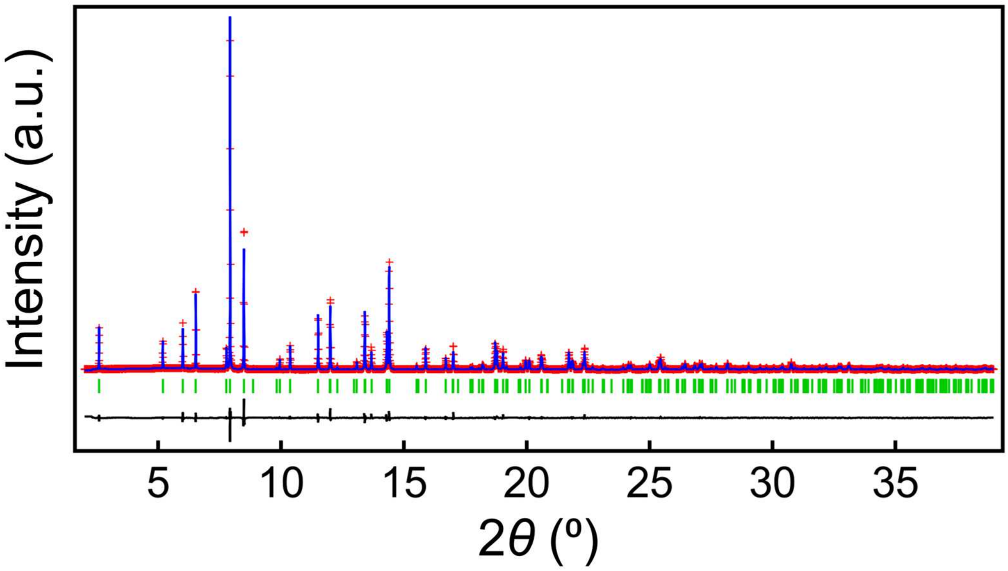

Figure S23. Rietveld pattern of $Bi_{1.9}Te_{0.1}LuO_{4.05}Cl$ for synchrotron X-ray powder diffraction data measured at 27 °C. Red crosses, blue solid line, and black solid line stand for the experimental data, calculated intensities, and difference pattern, respectively. Green vertical bars denote calculated Bragg peak positions of *P*4/*mmm* $Bi_{1.9}Te_{0.1}LuO_{4.05}Cl$.

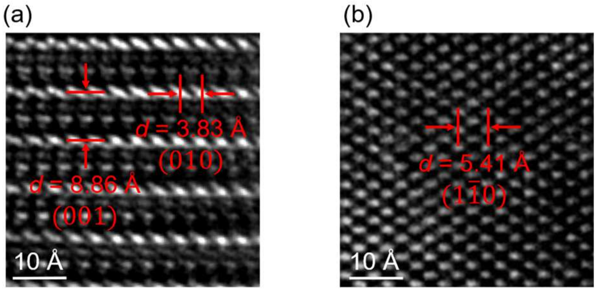

Figure S24. High-resolution transmission electron microscopy (HRTEM) images of $Bi_{1.9}Te_{0.1}LuO_{4.05}Cl$ along the (a) [100] and (b) [221] zone axes. The lattice spacings *d* shown in the panels (a) and (b) agree with those from the neutron diffraction data (Table S1).

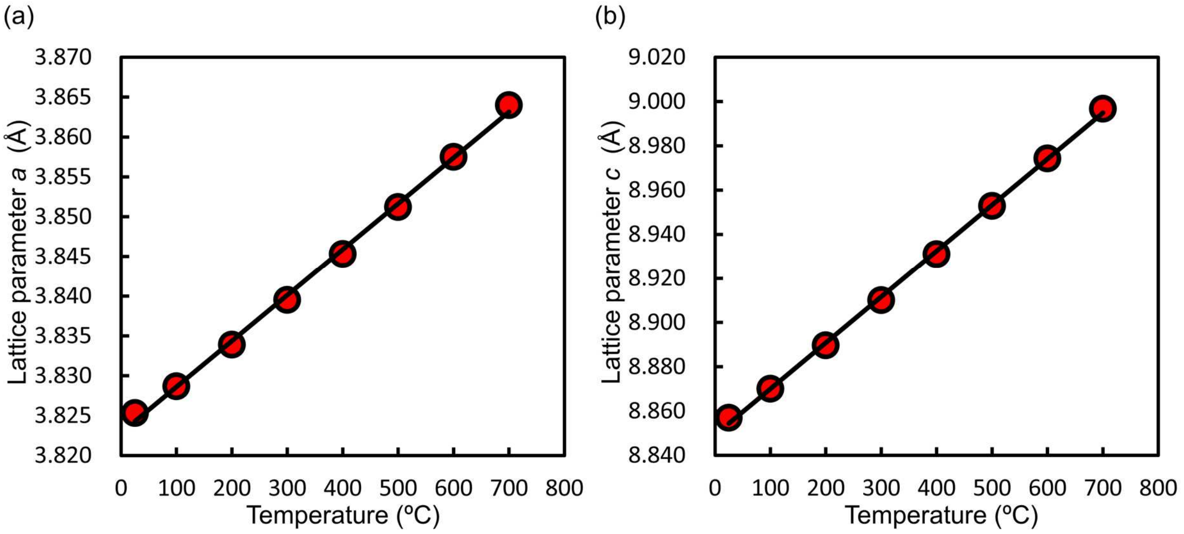

Figure S25. Lattice parameters (a) *a* and (b) *c* of $Bi_{1.9}Te_{0.1}LuO_{4.05}Cl$ as functions of temperature.

Supplementary Note S6.

The average thermal expansion coefficients (TECs) along the *a*- and *c*-axes between 25 and 700 °C are defined as

$$\alpha_a \equiv [a(700) - a(25)] \,/\, [a(25)(700 - 25)], \qquad (3)$$

$$\alpha_c \equiv [c(700) - c(25)] \,/\, [c(25)(700 - 25)]. \qquad (4)$$

Here *a*(*T*) and *c*(*T*) are the refined lattice parameters *a* and *c*, respectively, at temperature *T* (°C). The α_a and α_c were estimated to be $1.4983(5)\times10^{-5}$ K^{-1} and $2.3387(8)\times10^{-5}$ K^{-1}, respectively, at 25–700 °C. The average TEC of the cube root of the lattice volume between 25 and 700 °C is defined as

$$\alpha_{\sqrt[3]{V}} \equiv [\sqrt[3]{V(700)} - \sqrt[3]{V(25)}] \,/\, [\sqrt[3]{V(25)}(700 - 25)] \qquad (5)$$

Here *V*(*T*) is the lattice volume *a*(*T*)×*a*(*T*)×*c*(*T*) at temperature *T* (°C). The $\alpha_{\sqrt[3]{V}}$ was estimated to be $1.778(9)\times10^{-5}$ K^{-1} at 25–700 °C.

Supplementary Note S7. Crystal structures of Bi_2LuO_4Cl and $Bi_{1.9}Te_{0.1}LuO_{4.05}Cl$

Figures S26a and S26b show the refined crystal structures of (a) Bi_2LuO_4Cl and (b) $Bi_{1.9}Te_{0.1}LuO_{4.05}Cl$, respectively, which were obtained using neutron diffraction data taken at 25 °C.

(1) Crystal structure of Bi_2LuO_4Cl: There are four independent crystallographic sites (labels: Lu, Bi, O1, and Cl) in Bi_2LuO_4Cl. In Bi_2LuO_4Cl, the Lu atom is coordinated by 8 oxygen atoms (Figure S27a), while the Bi atom is coordinated by 4 oxygen atoms and 4 Cl atoms (Figure S27b). The crystal structure of Bi_2LuO_4Cl consists of a triple fluorite-like layer and a Cl layer (Figure S26a). The triple fluorite-like Bi_2LuO_4 layer is composed of two Bi and one Lu layers (three cation layers), and two O1 layers.

(2) Crystal structure of $Bi_{1.9}Te_{0.1}LuO_{4.05}Cl$: There are five independent crystallographic sites (labels: Lu, Bi/Te, O1, O2, and Cl) in the crystal structure of $Bi_{1.9}Te_{0.1}LuO_{4.05}Cl$. In $Bi_{1.9}Te_{0.1}LuO_{4.05}Cl$, the Lu atom is coordinated by 8.1 oxygen atoms (Figure S27c), while the Bi atom is coordinated by 4 oxygen atoms and 4 Cl atoms (Figure S27d). The crystal structure of $Bi_{1.9}Te_{0.1}LuO_{4.05}Cl$ consists of a triple fluorite-like layer and a Cl layer (Figure S26b). The triple fluorite-like $Bi_{1.9}Te_{0.1}LuO_{4.05}$ layer is composed of two Bi/Te and one Lu−O2 layers (three cation layers), and two O1 layers.

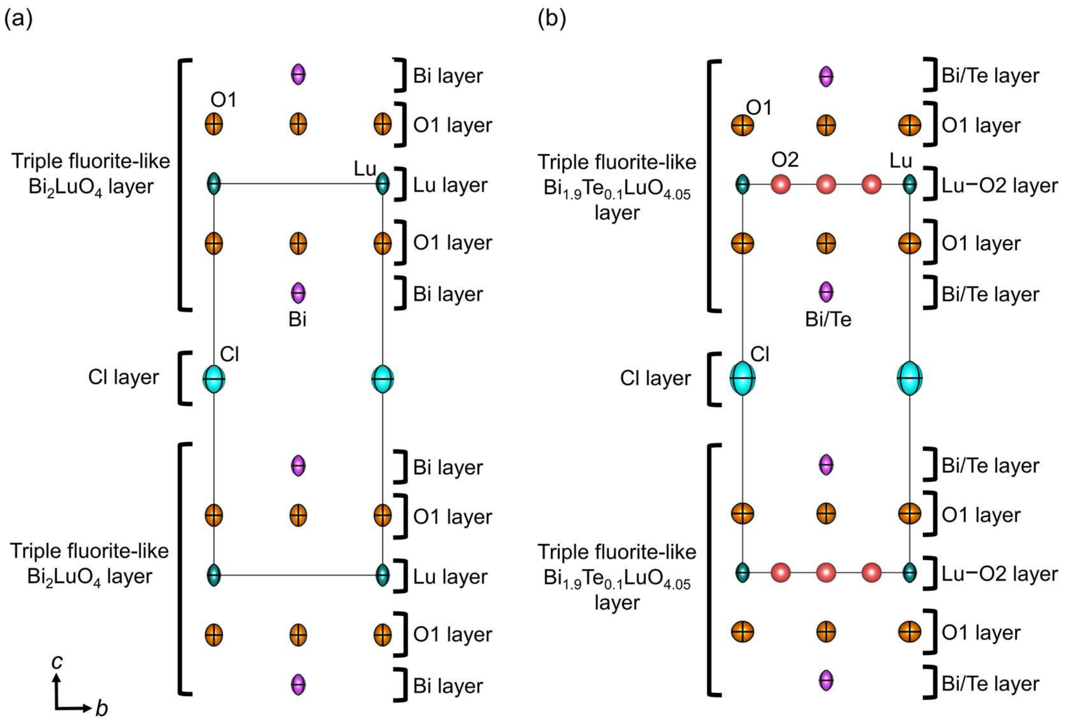

Figure S26. Refined crystal structures of (a) Bi_2LuO_4Cl at 25 °C and (b) $Bi_{1.9}Te_{0.1}LuO_{4.05}Cl$ at 25 °C, viewed along the *a*-axis ($0 \leq x \leq 1.0$; $0 \leq y \leq 1.0$; $-0.3 \leq z \leq 1.3$). Orange, purple, dark green, and light blue ellipsoids in (a) denote the O1, Bi, Lu, and Cl atoms, respectively. Orange, purple, dark green, and light blue ellipsoids in (b) denote the O1, Bi/Te, Lu, and Cl atoms, respectively. Red spheres in (b) denote interstitial O2 atoms. Thermal ellipsoids and spheres are drawn at the 75% probability level. The black rectangle represents the unit cell.

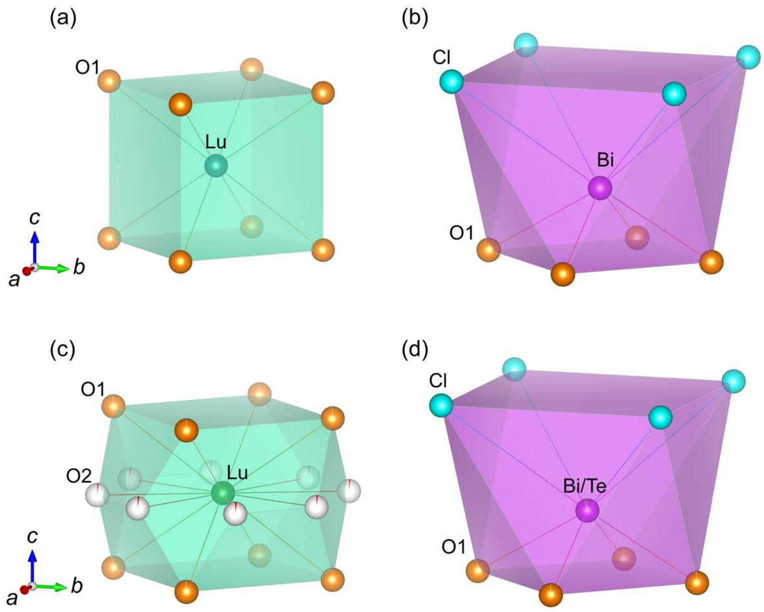

Figure S27. Coordination polyhedra of (a) LuO_8 and (b) BiO_4Cl_4 in Bi_2LuO_4Cl. Coordination polyhedra of (c) $LuO_{8.1}$ and (d) $(Bi/Te)O_4Cl_4$ in $Bi_{1.9}Te_{0.1}LuO_{4.05}Cl$. The coordination numbers of the Lu atom in $Bi_{1.9}Te_{0.1}LuO_{4.05}Cl$ (8.1) is higher than that in Bi_2LuO_4Cl (8.0).

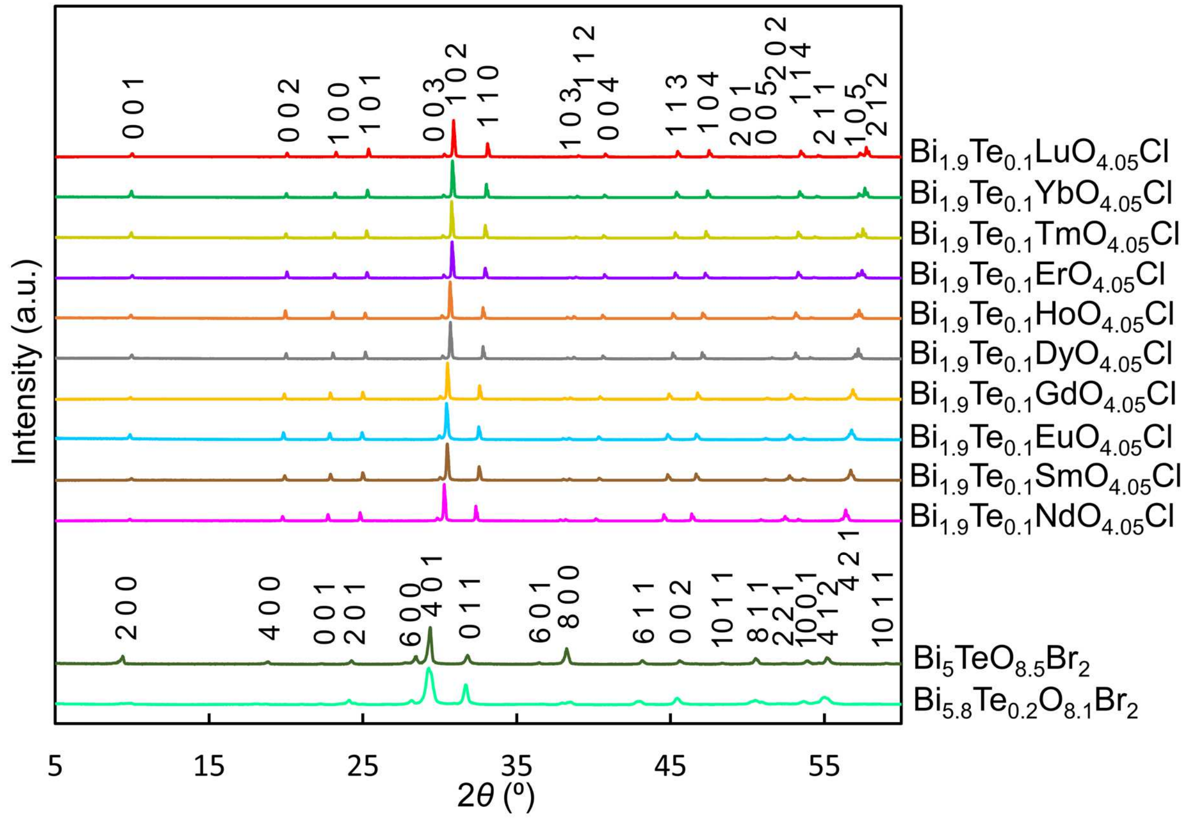

Figure S28. Cu Kα XRD patterns of $Bi_{1.9}Te_{0.1}RO_{4.05}Cl$ (*R* = Nd, Sm, Eu, Gd, Dy, Ho, Er, Tm, Yb, Lu) and $Bi_{6-2x}Te_{2x}O_{8+x}Br_2$ (x = 0.1, 0.5) samples at room temperature. *hkl* for $Bi_{1.9}Te_{0.1}RO_{4.05}Cl$ (*R* = Nd, Sm, Eu, Gd, Dy, Ho, Er, Tm, Yb, Lu) denotes the reflection index based on the primitive tetragonal lattice (*P*4/*mmm*), indicating the Sillén phase with the triple fluorite-like layer. *hkl* for $Bi_{6-2x}Te_{2x}O_{8+x}Br_2$ (x = 0.1, 0.5) denotes the reflection index based on the primitive tetragonal lattice (*Pmmm*), indicating the Sillén phase with the triple fluorite-like layer. Attempt to prepare Sillén phase $Bi_{1.9}Te_{0.1}ScO_{4.05}Cl$ (*R* = Sc) was unsuccessful due to the formation of impurities such as $Bi_4Cl_2O_5$.

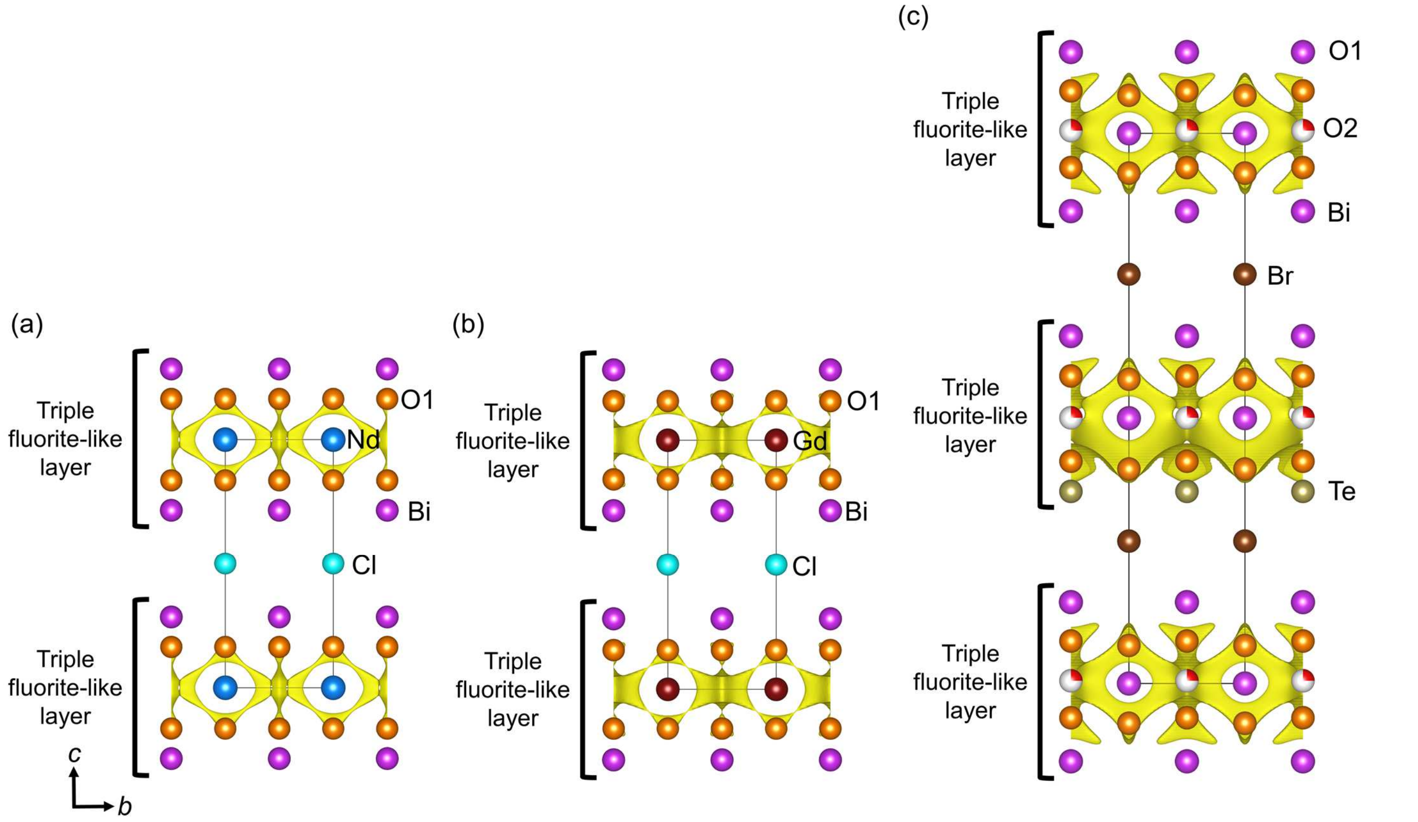

Figure S29. Yellow isosurfaces of BVE for a test oxide ion (a) at 0.22 eV of Bi_2NdO_4Cl,[9] (b) at 0.30 eV of Bi_2GdO_4Cl,[9] and (c) at 0.40 eV of $Bi_5TeO_{8.5}Br_2$,[10] which were projected on the *bc* plane. These BVE landscapes show two-dimensional oxide-ion diffusion in the triple fluorite-like layers.

Reference

[1] Irvine, J. T. S.; Sinclair, D. C.; West, A. R. Electroceramics: Characterization by Impedance Spectroscopy. *Adv. Mater.* **1990**, *2* (3), 132–138.

[2] Lepley, N.; Holzwarth, N. Computer Modeling of Crystalline Electrolytes - Lithium Thiophosphates and Phosphates. *ECS Trans.* **2011**, *35* (14), 39–51.

[3] Du, Y. A.; Holzwarth, N. A. W. Mechanisms of Li^+ diffusion in crystalline γ- and β-Li_3PO_4 electrolytes from first principles. *Phys. Rev. B* **2007**, *76* (17), 174302.

[4] Du, Y. A.; Holzwarth, N. A. Li ion migration in Li_3PO_4 electrolytes: Effects of O vacancies and N substitutions. *ECS Trans.* **2008**, *13* (26), 75–82.

[5] Taninouchi, Y.; Uda, T.; Ichitsubo, T.; Awakura, Y.; Matsubara, E. High oxide-ion conductivity of monovalent-metal-doped bismuth vanadate at intermediate temperatures. *Solid State Ion.* **2010**, *181* (15–16), 719–723.

[6] Yamamura, H.; Higasa, M.; Yagi, Y.; Takayama, T. Electrical conductivity in the $(Bi_{0.8-x}Ln_xEr_{0.2})_2O_3$ (*Ln* = La, Pr, Nd) systems. *J. Ceram. Soc. Jpn.* **2009**, *117* (8), 887–890.

[7] Guillodo, M.; Fouletier, J.; Dessemond, L.; Del Gallo, P. Electrical properties of dense Me-doped bismuth vanadate (Me=Cu, Co) pO_2-dependent conductivity determined by impedance spectroscopy. *J. Eur. Ceram. Soc.* **2001**, *21* (13), 2331–2344.

[8] Yaguchi, H.; Morikawa, D.; Saito, T.; Tsuda, K.; Yashima, M. High Oxide-Ion Conductivity through the Interstitial Oxygen Site in Sillén Oxychlorides. *Adv. Funct. Mater.* **2023**, *33* (27), 2214082.

[9] Schmidt, M.; Oppermann, H.; Hennig, C.; Henn, R. W.; Gmelin, E.; Söger, N.; Binnewies, M. Untersuchungen zu Bismutseltenerdoxidhalogeniden der Zusammensetzung Bi_2SEO_4X (X = Cl, Br, I). *Z. Anorg. Allg. Chem.* **2000**, *626* (1), 125–135.

[10] Dolgikh, V.A.; Kholodkovskaya, L.N.; Popovkin, B.A. Crystal Structure of $Bi_5TeO_{8.5}Br_2$: Coordination of Te(IV) Atoms in Layered Sillén Phases. *Russ. J. Inorg. Chem.* **1996**, *41* (6), 932–936.
